# Leveraging Piezoelectric and Ferroelectric Effects to Control Zinc Deposition for High-Performance Solid-State Zinc Batteries

**DOI:** 10.1021/jacs.5c23299

**Published:** 2026-04-16

**Authors:** Yue Hou, Qiong Liu, Zeru Wang, Xinru Yang, Dedi Li, Yiqiao Wang, Zhiquan Wei, Zhaodong Huang, Qing Li, Ke Wang, Chunyi Zhi

**Affiliations:** † Hong Kong Center for Cerebro-Cardiovascular Health Engineering (COCHE), Shatin, New Territories, Kowloon, Hong Kong 999077, China; ‡ School of System Design and Intelligent Manufacturing, 255310 Southern University of Science and Technology, Shenzhen 518055, China; § Department of Materials Science and Engineering, 53025 City University of Hong Kong, 83 Tat Chee Avenue, Kowloon, Hong Kong 999077, China; ∥ College of Physics, 12530 Sichuan University, Chengdu, Sichuan 610065, China; ⊥ Institute of Applied Physics and Materials Engineering, 59193 University of Macau, Macau 999078, China; # Department of Mechanical Engineering, 25809 The University of Hong Kong, Pokfulam, Kowloon, Hong Kong 999077, China; ∇ Materials Innovation Institute for Life Sciences and Energy (MILES), HKU-SIRI, Shenzhen 518048, China; ○ Center for Energy Storage, The University of Hong Kong, Pokfulam, Kowloon, Hong Kong 999077, China; ◆ Department of Chemical and Biological Engineering, Hong Kong University of Science and Technology, Clear Water Bay, Kowloon, Hong Kong 999077, China

## Abstract

Solid electrolytes with piezoelectric and ferroelectric properties can form stable interface structures through spontaneous polarization under electrostatic potential differences. The presence of a ferroelectric polarization electric field can reduce the initial electrostatic potential difference and minimize adverse ion aggregation in the electrical double layer (EDL). Herein, we integrated piezoelectric and ferroelectric CaBi_2_Nb_2_O_9_ (CBN) sheets into a solid polymer electrolyte (SPE) based on a poly­(vinylidene difluoride) (PVDF) matrix, which is referred to as CBN@PVDF. Experimental results and theoretical simulations reveal that the piezoelectric effect of the CBN, induced by mechanical stress during zinc plating, can diminish the driving force for dendrite growth in regions of high curvature. Simultaneously, its ferroelectric properties can lower the local overpotential, resulting in even deposition of Zn. As expected, the symmetric Zn|CBN@PVDF|Zn batteries exhibit unprecedented cycling stability, achieving lifespans of 2000 h at 0.5 mA cm^–2^, and 1500 h at 1.0 mA cm^–2^, respectively. In addition, incorporating CBN could enhance the dielectric properties of the SPE, improve salt dissociation, and increase the ionic conductivity of the SPE, thereby achieving a superior rate performance for Zn||pyrene-4,5,9,10-tetraone (PTO) solid full cells. It can function at an exceptionally high rate of 10 C, achieving a high specific capacity of 221 mAh g^–1^. Overall, designing piezoelectric/ferroelectric SPEs can effectively address the challenges of nonuniform Zn deposition and low ionic conductivity of SPE, providing a robust foundation for the development of high-performance solid-state zinc batteries.

## Graphical abstract

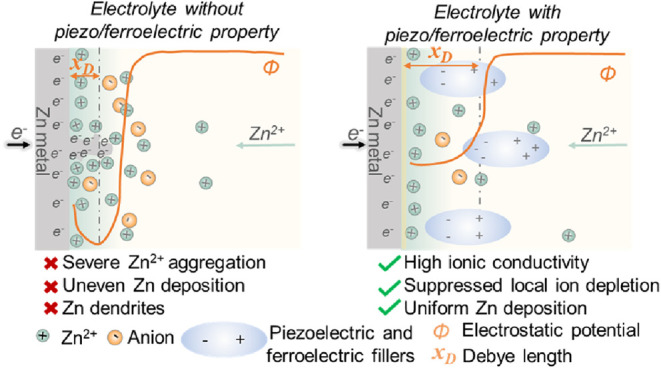

## 1 Introduction

Solid-state zinc ion batteries (SSZIBs) have shown great promise as the next generation of low-cost and intrinsically safe battery technology for portable energy suppliers and extensive urban power networks.^1–3^ They integrate the merits of solid electrolytes, such as the wide electrochemical window, alleviation of hydrogen evolution reaction (HER) issues in aqueous electrolytes, suppressed Zn dendrites, etc.^4–7^ However, uncontrolled Zn metal dissolution can still cause dead Zn accumulation at the anode side, hampering the extended durability and exceptional safety sought in SSZIBs. Solid electrolytes usually exhibit a poorer critical current density when compared with aqueous electrolytes due to their poorer interfacial compatibility. This feature would result in the formation and expansion of anisotropic Zn filaments.

In classical electrochemical theory, there is a dynamic competition between the dissolution of Zn metal and the deposition of Zn^2+^ ions.^8–10^ The imbalance of competition leads to the growth of Zn dendrites. Specifically, the Chazalviel model suggests that dendrites form due to the unequal movement of cations and anions in an electrolyte.^11,12^ It is predicted that a lack of anions near the Zn electrode creates a polarized electric field, which can promote dendrite growth on the Zn metal surface.^11–13^ Electrolytes having high ionic conductivity and low mobility of anions will slow down dendrite generation by reducing the depletion of anions at electrode–electrolyte interface.^14,15^ Thus, conventional protocols to control Zn dissolution kinetics and further reduce the Zn dendrite formation in SSZIBs include the increase the ionic conductivity of solid electrolytes,^16,17^ improving and balancing the electronic conductivity at the electrodes,^18,19^ and building a seamless interface between electrode and solid electrolytes.^20,21^ The strategies mentioned above are useful to balance the unequal Zn deposition dynamics. However, activation overpotential is the decisive driving force to promote Zn dendrite propagation.^22,23^ The irregular electrodeposition and the dendrite generation would be induced by large activation overpotential.^24,25^

To circumvent Zn deposition, some researchers have proposed introducing an additional electric field between the solid electrolyte and the anode to decrease the activation potential. Figure 1a,b illustrates the electrical double layer (EDL) of the Zn-solid electrolyte interface at the initial stage and during electrodeposition, with and without piezoelectric and ferroelectric effects, respectively. This illustration is predicated on the fundamental understanding of EDL behavior. Given that the spatial scale of the EDL is comparable to the Debye length $x_{D}={\left(\frac{\mathrm{RT}}{2F^{2}c_{\mathrm{bulk}}}\varepsilon_{0}\right)}^{1/2}$ , where *R* denotes the gas constant, *T* represents the absolute temperature, *F* stands for the Faraday constant, *c*_bulk_ is the bulk concentration, and ε_0_ is the vacuum permittivity, the Poisson equation governs the spatial distribution of the electrostatic potential (ϕ).^26^ Research on the EDL based on ion, hole, and electron equilibrium indicates that the electrostatic potential of solid electrolytes is typically higher than that of the Zn anode for most Zn-solid electrolyte interfaces.^27–31^ During the electrodeposition process, a large overpotential caused by the slow diffusion of Zn^2+^ ions and an uneven distribution of Zn^2+^ concentration could cause rapid growth of Zn dendrites and battery failure (Figure 1a). In contrast, as illustrated in Figure 1b, the piezoelectric and ferroelectric properties of solid electrolytes can create a more stable interface structure through spontaneous polarization under electrostatic potential differences. The presence of a ferroelectric polarization electric field can reduce the initial electrostatic potential difference and suppress adverse ion aggregation in the EDL.^32,33^ Piezo-/ferroelectric fillers could also enhance the dielectric properties of solid electrolytes. Compared with conventional fillers, this enhancement leads to a larger Debye length. Consequently, charges near the interfacial region become more dispersed, which requires a longer distance for charge depletion. This expanded spatial charge distribution promotes a more uniform concentration of ions at the electrode surface, mitigating local ion depletion that is widely considered a primary cause of dendritic protrusions. By reducing the potential difference and enlarging the Debye length, ferroelectric fillers could suppress local ion depletion and promote uniform ionic flux, thereby inhibiting dendrite initiation and ensuring stable battery cycling.

**Figure 1:**
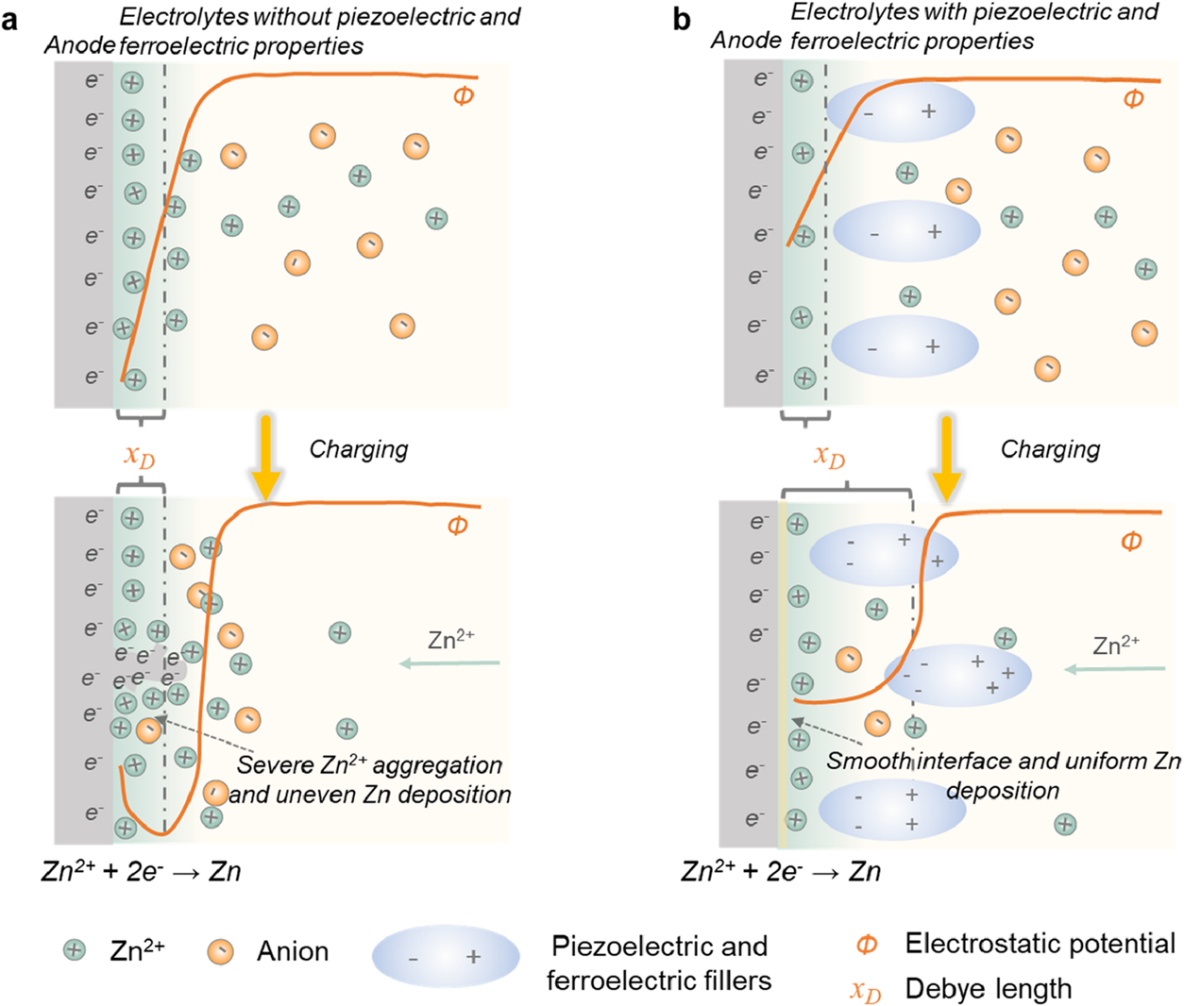
Schematic EDL in the interface between Zn metal and (a) electrolytes without piezoelectric and ferroelectric properties at the initial state and during electrodeposition or (b) electrolytes with piezoelectric and ferroelectric properties at the initial state and during electrodeposition. The EDL is formed when solvated Zn^2+^ ions in electrolytes counterbalance the excess electrons on the Zn metal.

In this paper, we incorporate piezoelectric and ferroelectric CaBi_2_Nb_2_O_9_ (CBN) sheets into a poly­(vinylidene difluoride) (PVDF)-based solid polymer electrolyte (SPE) to ensure a uniform electric field distribution within the SPE. Incorporating CBN could also enhance the dielectric properties of the SPE, further improving salt dissociation. This would increase the number of ionic clusters, thereby enhancing the ionic conductivity of the SPE. The stress generated during zinc deposition can initiate the piezoelectric and ferroelectric effect of CBN, thereby reducing the local overpotential, regulating the even distribution of Zn^2+^, and thus achieving high reversibility and an unprecedented stable cycle life for SSZIBs.

## 2 Results and Discussion

### 2.1 Characterization of CBN and CBN@PVDF

The piezoelectric effect in the CBN enables the generation of an electric field when a mechanical force is applied to the material.^34^ This phenomenon allows for the efficient separation of electron–hole pairs owing to the induced electric field. These distinctive properties of CBN render it an up-and-coming candidate for effectively manipulating the electric field within SPEs. CBN was prepared via a molten-salt process. The scanning electron microscopy (SEM) image (Figure S1a) and the high-resolution transmission electron microscopy (HRTEM) image (Figure S1b) display that the structure of CBN is relatively uniform and in a layered form. As shown in Figure S1c, the HRTEM image of CBN reveals lattice fringe distances of 0.273 and 0.275 nm, assigned to the (020) and (200) planes, respectively. This indicates that the dominant exposed facets are (00l)-oriented, reflecting a high degree of crystallinity.^35^ Then, we simply mixed the CBN with PVDF and zinc trifluoromethanesulfonate (Zn­(OTf)_2_) to fabricate the PVDF-based SPE, in which the weight ratios of CBN were adjusted from 0 to 15 wt %. To screen the most suitable SPE with a high ionic conductivity, titanium (Ti)|SPE|Ti cells were assembled and tested at ambient temperature (Figure S2a). The SPE with 10 wt % CBN presents the highest ionic conductivity (1.04 mS cm^–1^) among all of the samples (Figure S2b). Thus, the SPE containing 10 wt % CBN was identified as the optimized composition and designated as CBN@PVDF. For comparison, the control SPE containing no CBN is labeled as PVDF.

As presented by the top-view and cross-sectional SEM images of CBN@PVDF (Figure S3a,b), the as-prepared CBN@PVDF exhibits a compact and uniform morphology. The membrane thickness was measured to be 74.6 ± 5.36 μm, corresponding to a relative variability of 7.18%. The height topology map of atomic force microscopy (AFM) (Figure S4) was obtained to identify the surface morphology of CBN@PVDF. The AFM image shows that CBN@PVDF exhibits a smooth, relatively uniform surface, consistent with the SEM results. The dense and uniform surface of CBN@PVDF can achieve tight interface contact between electrodes. In addition, as observed from Figure S5, the components of CBN@PVDF were detected by X-ray diffraction (XRD). The CBN powder presented a two-layer Aurivillius-type structure with an orthorhombic *A*2_1_ *am* space group.^36,37^ This structure was found to conform to the standard PDF card (No. 49–0608). The diffraction peaks located at 14.2°, 25.4°, 29.2°, 29.4°, 32.6°, 36.1°, 49.6°, 60.7°, 68.4°, and 76.3° correspond to the (004), (113), (008), (115), (200), (0010), (0210), (2210), (400), and (048) crystal plane of the CBN lattice.^35^ The three distinct peaks at (004), (008), and (0010) indicate that the dominant exposed facets are (00l)-oriented, consistent with the HRTEM results. In Figure S5, the XRD pattern of CBN@PVDF shows a sharp diffraction peak at 18.5°, assigned to the (020) crystal plane of the α-phase PVDF, alongside a broad peak at 20.6°, which arises from the (110) crystal plane of the β-phase PVDF. This pattern also demonstrates that the incorporation of PVDF did not alter the crystalline structure or phase of CBN, and further confirms the homogeneous dispersion of CBN within the as-prepared SPE. Fourier transform infrared spectroscopy (FTIR) was also employed to demonstrate the successful synthesis of CBN@PVDF, wherein the apparent stretching vibration modes of Bi–O3 peaks at 423 cm^–1^, Nb–O peaks at 570 cm^–1^, and Nb–O5 peaks at 801 cm^–1^ from CBN appear in the CBN@PVDF (Figure S6).^37^ The combined XRD and FTIR analyses thus provide solid evidence for the successful fabrication of the CBN@PVDF composite.

As shown in Figure S7, the CBN@PVDF exhibits a higher yield strength (10.13 MPa) than that of PVDF. This improvement in yield strength is accompanied by a reduction in plastic deformation capacity, attributed to mechanical constraints and stress concentrations induced by rigid inorganic CBN fillers. Specifically, the inherent mismatch in mechanical properties between the soft PVDF matrix and the hard CBN filler promotes interfacial debonding and cavitation under stress, thereby reducing the plastic deformation capacity of the PVDF SPE.^38–41^ Nevertheless, this trade-off in ductility is offset by the gain in mechanical strength. The enhanced yield strength of CBN@PVDF plays a pivotal role in suppressing dendrite growth by providing a higher mechanical barrier, thereby improving cycle stability of the SSZIBs. Moreover, as evidenced by the broadband dielectric spectroscopy results in Figure S8, the dielectricity of PVDF-based SPE is promoted by the incorporation of CBN, which is beneficial to the dissociation of Zn­(OTf)_2_ within SPE.

Piezo-/ferroelectric properties of CBN were analyzed using piezoresponse force microscopy (PFM). It can be observed in Figure 2a that the CBN sheets present an apparent mechanical displacement when the applied bias changes. The CBN platelets exhibit a surface potential difference of approximately 0.82 V, as the crystal growth of CBN aligns along the (001) direction. When subjected to an applied force, the dipoles within the platelets undergo a slight movement in the *x*–*y* plane, generating a small built-in electric field.^42^ This spontaneously polarized electric field offers a means to reduce the local overpotential. Figure 2b illustrates an amplitude curve in the shape of a butterfly when the applied voltage varied from −10 V to +10 V. The distinct butterfly loop confirms the strong regional piezoelectric response of CBN sheets. Moreover, as shown in the phase-hysteresis loop (the blue line in Figure 2b), the phase angles shift from −40 to 150° under a 10 V DC bias field, suggesting the presence of nonzero remanent polarization and confirming the ferroelectric nature of CBN. It demonstrates that the polarization reverses when the electric field direction is reversed.^43,44^ Furthermore, the piezo-/ferroelectric signals of CBN@PVDF were also detected by PFM (Figures 2c and S9a,b). The phase image acquired at a bias of 0.5 V for the fabricated CBN@PVDF (Figure 2c) further proves that CBN gives a response to the varied electric field and undergoes phase reversal, although it is embedded within the PVDF. Moreover, the PFM amplitude result recorded at a bias of 0.5 V for CBN@PVDF (Figure S9b) indicates that introducing CBN allows the CBN@PVDF to exhibit a robust feedback electrical signal. Then, we analyzed the Kelvin probe force microscopy (KPFM) results to prove the electric field effects on the ion transport mechanism.^45^ The mean interfacial potential for PVDF is 22 mV (Figure 2d,e) and obviously larger than that of CBN@PVDF (9.0 mV; Figure 2f,g), suggesting that the inclusion of CBN can effectively reduce the overpotential at the electrode–electrolyte interface, thereby lowering the energy barriers of ion migration. Consequently, the piezo-/ferroelectric CBN can generate a built-in electric field, mitigating the detrimental aggregation of ions in the EDL.

**Figure 2:**
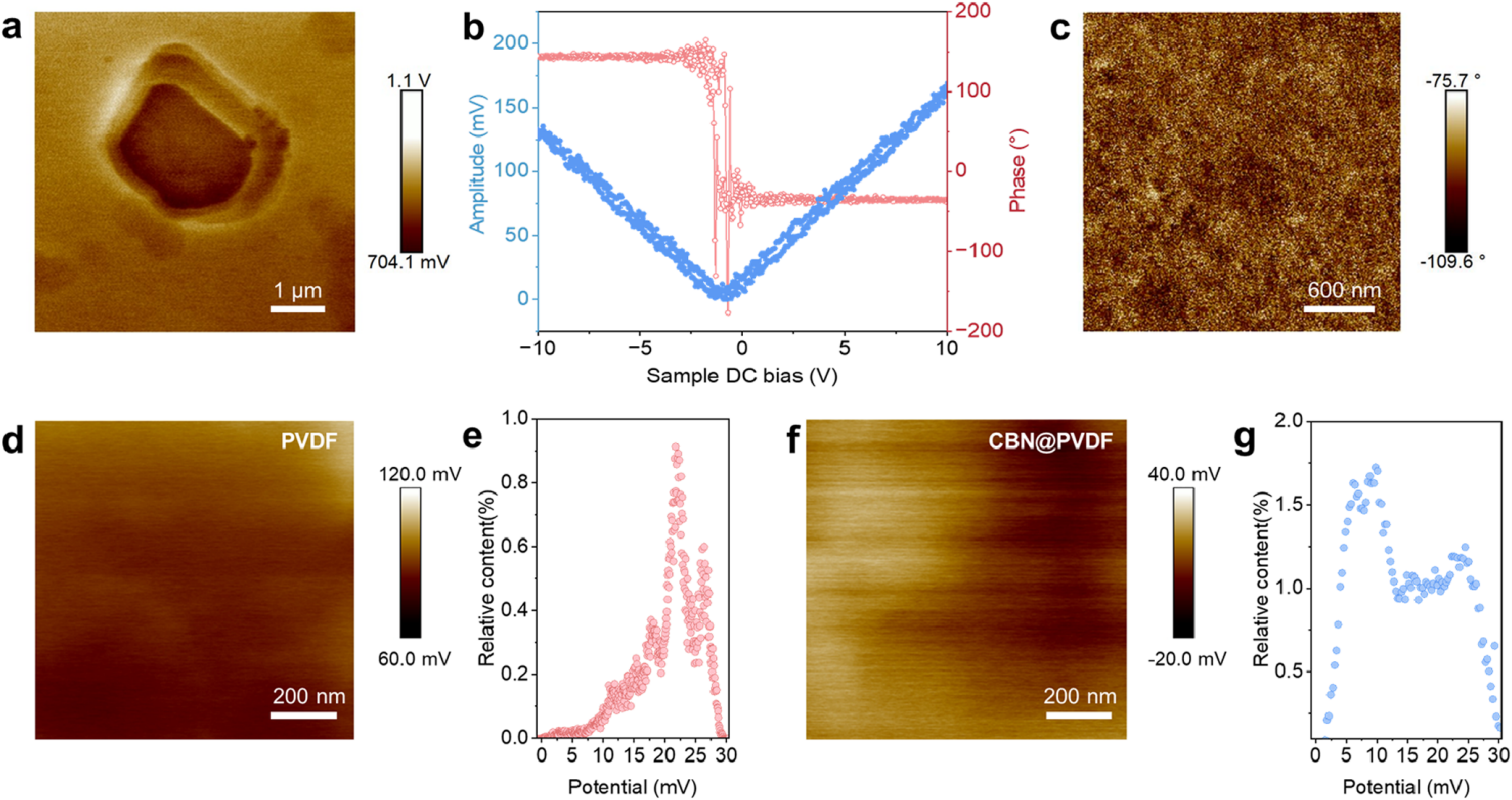
(a) Surface potential obtained from PFM measurements of the CBN. (b) Amplitude/phase–voltage loop of CBN. (c) PFM phase image for CBN@PVDF at a bias of 0.5 V. (d) KPFM interfacial potential images of the interfaces between PVDF and Zn with its corresponding Gauss statistic distribution histogram (e). (f) KPFM interfacial potential images of the interfaces between CBN@PVDF and Zn, with its corresponding Gauss statistic distribution histogram (g).

### 2.2 Ion Migration and Zn Anode Reversibility

The differential scanning calorimetry (DSC) curves in Figure S10 reveal that the glass transition temperature (*T*_g_) of CBN@PVDF is −34.2 °C, which is lower than that of the PVDF. The addition of CBN can increase the amorphous region of the PVDF matrix, contributing to accelerating the movement of polymer segments. Thus, fast ion transportation is expected at ambient temperature for CBN@PVDF. To validate this, we measured the ionic conductivities of PVDF and CBN@PVDF across a broad temperature range from 25 to 85 °C by analyzing their EIS results (Figures 3a and S11). It was found that the ionic conductivities of these SPEs increase with temperature, which is attributed to the improved mobility of polymer segments at high temperatures. Additionally, the activation energy (*E*_a_) of the as-prepared SPEs is calculated using the Arrhenius equation.^46^ The activation energy (*E*_a_) for ion migration within CBN@PVDF decreases from 0.030 eV (PVDF) to 0.014 eV by introducing CBN (Figure 3a). Based on these results, the incorporation of CBN in SPEs effectively reduces ion aggregation and enhances charge mobility, resulting in a significant improvement in ionic conductivity. We also conducted further chronoamperometry tests to calculate the transference number of PVDF (Figure S12a,b) and CBN@PVDF (Figure S12c,d). As summarized in Figure 3b, a high *t*_ion_ up to 0.55 is achieved by CBN@PVDF, while the *t*_ion_ of PVDF is only 0.20. It depicts the existence of abundant free Zn^2^ cations in CBN@PVDF. In addition, CBN@PVDF displays an ambient ionic conductivity of 1.04 mS cm^–1^, surpassing that of most prior studies on SPEs (Figure 3c). This outstanding performance is attributed to the high ε_r_ of CBN@PVDF, which favors salt dissociation and facilitates charge mobility, thereby significantly improving the ionic conductivity. Given these advantages, the exceptional ionic conductivity, rapid ion transport, and satisfactory *t*_ion_ achieved by the CBN@PVDF can facilitate the fast and uniform diffusion and deposition of Zn^2+^ during an electrochemical process.

**Figure 3:**
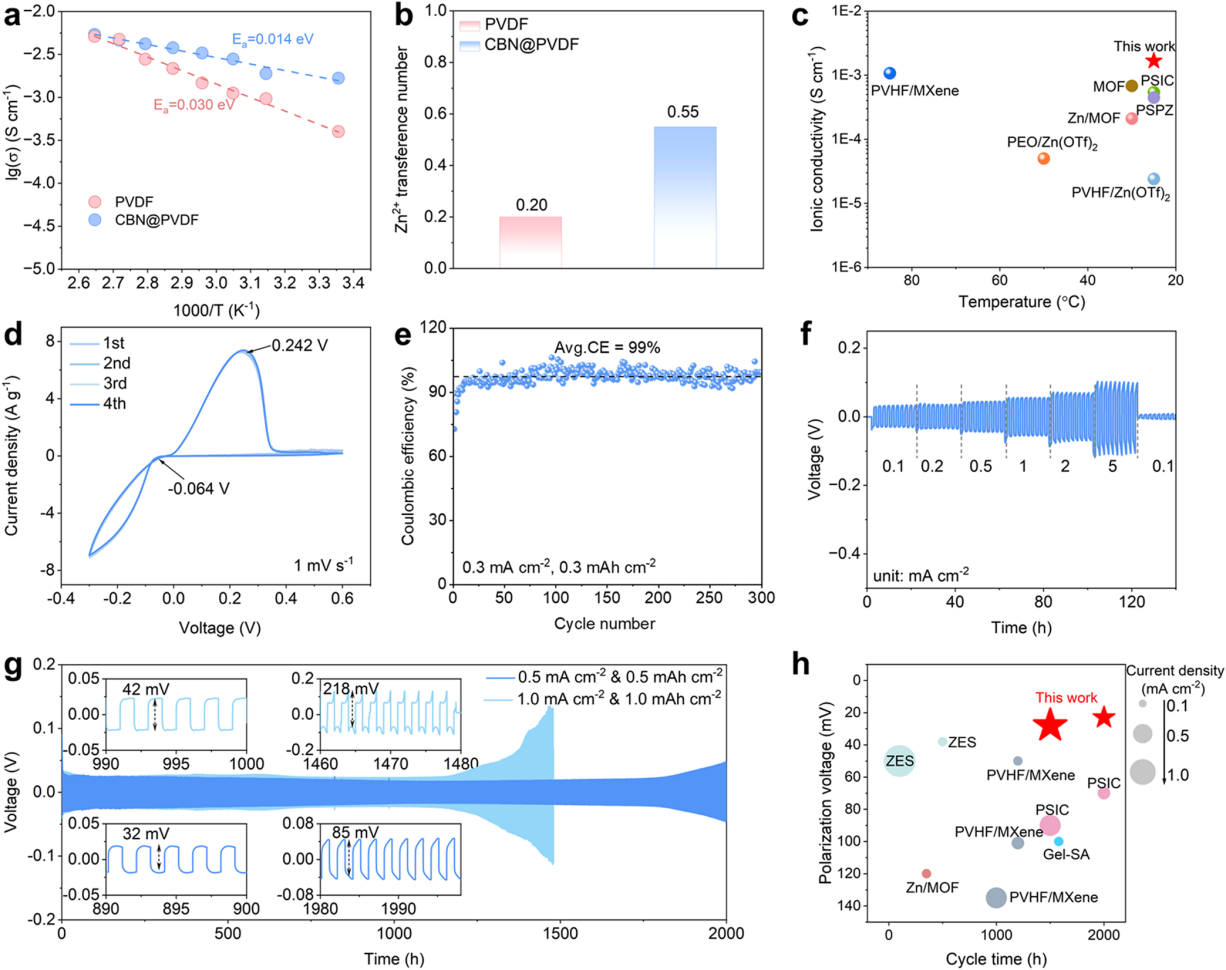
(a) Arrhenius plots for PVDF and CBN@PVDF. σ represents ionic conductivity, and *T* represents temperature. (b) Summary of Zn^2+^ transference number for PVDF and CBN@PVDF. (c) Benchmarking the ionic conductivity of this work against other studies (Table S2).^47–53^ (d) CV patterns of Zn plating/stripping. (e) Cycle tests of the Zn|CBN@PVDF|Ti cells at 0.3 mA cm^–2^ when the specific capacity is 0.3 mAh cm^–2^. (f) Voltage profiles of the Zn|CBN@PVDF|Zn cells recorded at different current densities. (g) Long-term cycling performances of the Zn|CBN@PVDF|Zn cells at 0.5 mA cm^–2^ with 0.5 mAh cm^–2^ and 1.0 mA cm^–2^ with 1.0 mAh cm^–2^. (h) Comparison of the cycle lifespan of Zn|CBN@PVDF|Zn cells with other previous literature (Table S3).^47,48,52,54,55^

Cyclic voltammetry (CV) measurements were employed to assess the plating/stripping reversibility of the Zn|CBN@PVDF|Ti batteries (Figure 3d). The results indicate that the onset potential of Zn^2+^ deposition is around −0.064 V (vs Zn^2+^/Zn), while the Zn dissolution potential is around 0.242 V (vs Zn^2+^/Zn). Notably, the first four CV patterns exhibit significant overlap, demonstrating the remarkable reversibility achieved by CBN@PVDF. The Zn|CBN@PVDF|Ti battery sustained stable operation over 300 cycles with an impressive Coulombic efficiency (CE) of approximately 99% (Figure 3e). This performance clearly surpasses many Zn||Ti batteries that employ aqueous electrolytes, which are often plagued by HER and susceptibility to short circuit. Its galvanostatic charge/discharge (GCD) curves also confirm the highly reversible Zn plating/stripping process (Figure S13). The CE gradually increases during the first 20 cycles and remains stable at around 99%, accompanied by a small Zn nucleation overpotential of 87 mV. These results imply enhanced interfacial compatibility between electrodes and the CBN@PVDF.

The cycling performances of Zn|CBN@PVDF|Zn batteries were measured under varied current densities from 0.1 to 5.0 mA cm^–2^ (Figure 3f). Minimal voltage hysteresis is observed in Zn|CBN@PVDF|Zn batteries, which points to the superior ionic conductivity of CBN@PVDF. Furthermore, the stability of CBN@PVDF was also investigated by carrying out long-term cycle tests on Zn||Zn symmetric batteries. These batteries achieve stable cycling for 2000 and 1500 h at 0.5 mA cm^–2^ and 1.0 mA cm^–2^, respectively (Figure 3g). In contrast, the Zn|PVDF|Zn battery maintained only a 700 h cycle life at 0.5 mA cm^–2^ (Figure S14). Specifically, the overpotential of the Zn|CBN@PVDF|Zn cell is smaller than that of the Zn|PVDF|Zn cell (96 mV), reaching 85 mV even after 2000 h cycling. These results highlight the superior reversibility of the Zn plating/stripping process obtained by our fabricated CBN@PVDF. To eliminate the probability of the “soft shorts,”^56^ EIS measurements were conducted at different charge conditions during the long-term cycle tests of Zn|CBN@PVDF|Zn batteries at 0.5 mA cm^–2^ and 1.0 mA cm^–2^. As observed from Figure S15a, the corresponding charge transfer resistance decreases from 450 to 76 Ω and remains stable throughout the 2000 h cycling period at 0.5 mA cm^–2^, as indicated by Nyquist plots fitted with the equivalent circuit. In contrast, a higher impedance is observed after 1500 h of cycling at 1.0 mA cm^–2^ (Figure S15b), decreasing from 1700 to 500 Ω. Then, the cycle time and polarization voltage at varied current densities of the Zn|CBN@PVDF|Zn symmetric batteries were compared with those of other works in Figure 3h. They show a minimal polarization voltage even at high current densities, which is ascribed to the outstanding stability of the Zn anode enabled by the piezo-/ferroelectric effects of our fabricated CBN@PVDF.

### 2.3 Piezo-/Ferroelectric Effects on Inhibiting Zn Dendrite Growth

To elucidate the piezo-/ferroelectric effect of CBN@PVDF on the Zn anode, SEM images were employed to monitor the Zn plating/stripping process. The custom-designed Zn|PVDF|Zn and Zn|CBN@PVDF|Zn cells were cycled under a current density of 0.5 mA cm^–2^ with a specific capacity of 0.5 mAh cm^–2^. As depicted by Figure 4a, the anode surface in the Zn|CBN@PVDF|Zn battery remains consistently flat without any formation of dendrite-like structures after 50 cycles, certifying that Zn^2+^ is deposited uniformly on the Zn anode. In contrast, island-like Zn dendrites are observed in the Zn|PVDF|Zn battery. As the current density increases, these island-dendrites turn into nanosheets (after 50 cycles) and readily bring about short-circuiting in the Zn|PVDF|Zn battery. This phenomenon is further corroborated by the laser confocal microscopy (LSM) three-dimensional (3D) images (Figure 4b). The LSM images reveal uniform Zn deposition for CBN@PVDF, whereas large aggregates and a nonuniform deposition surface are evident on the Zn anode with PVDF. These results suggest that CBN@PVDF facilitates uniform Zn deposition and stripping while maintaining a flat surface.

**Figure 4:**
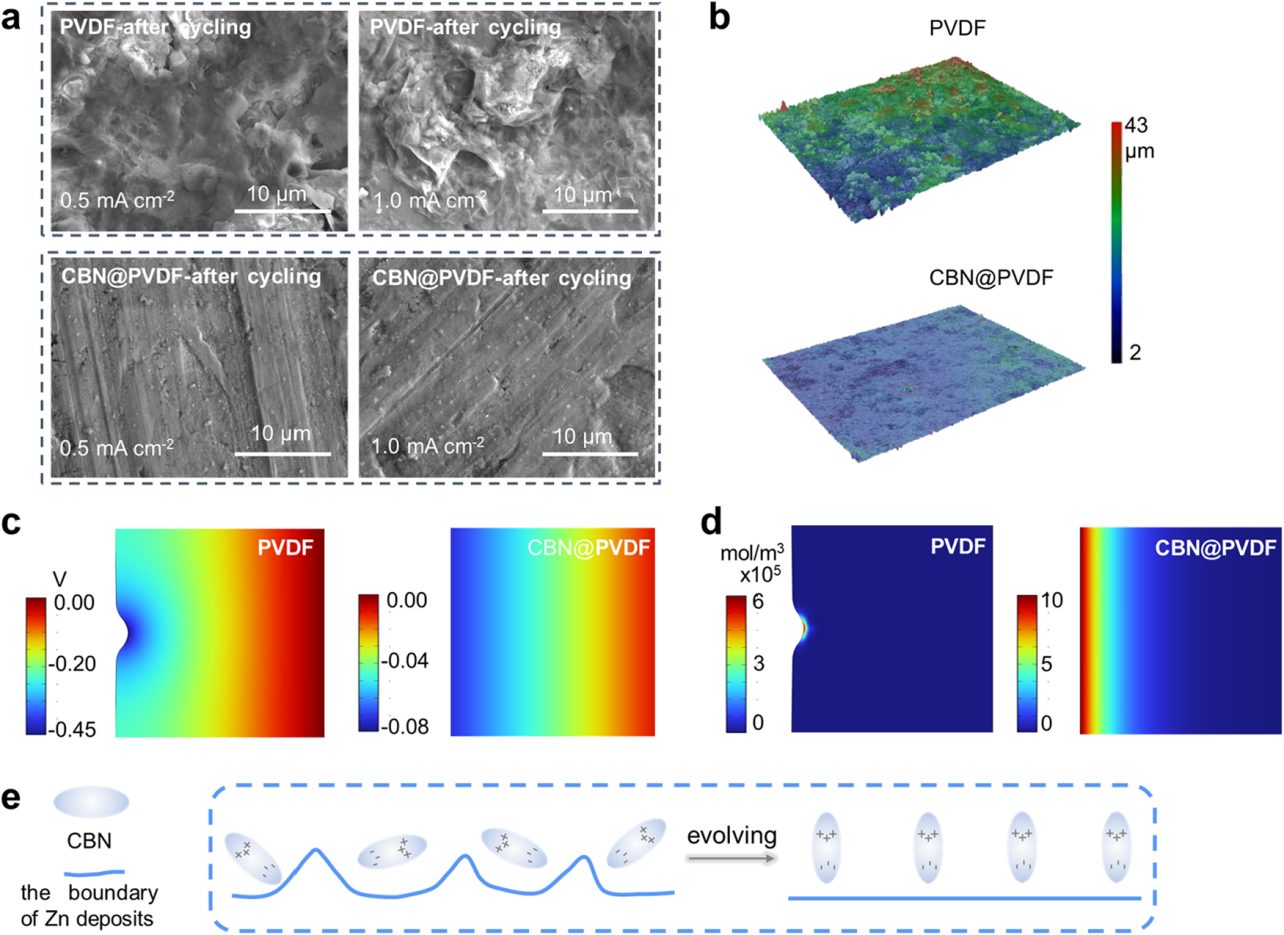
(a) SEM images of the Zn anode for Zn|PVDF|Zn and Zn|CBN@PVDF|Zn batteries at 0.5 mA cm^–2^ with 0.5 mAh cm^–2^ and 1.0 mA cm^–2^ with 1.0 mAh cm^–2^ after 50 cycles. (b) 3D LSM images of the Zn anode cycled in Zn|PVDF|Zn and Zn|CBN@PVDF|Zn cells at 0.5 mA cm^–2^ with 0.5 mAh cm^–2^ after 50 cycles. (c) Potential distribution simulated by COMSOL Multiphysics of the PVDF and CBN@PVDF. (d) Zn^2+^ concentration simulated by COMSOL Multiphysics in the PVDF and CBN@PVDF at 1 mA cm^–2^, respectively. (e) Schematic representation elucidating piezoelectric/ferroelectric effects when incorporating CBN fillers.

Furthermore, we conducted COMSOL Multiphysics simulations using a phase-field model that incorporates ionic conductivity, as well as piezo-/ferroelectric properties, to elucidate the morphological transition of Zn deposits resulting from weak solid-state interactions and dispersed forces with PVDF and CBN@PVDF, respectively. Figure 4c presents the two-dimensional snapshots of the electrostatic potential for the PVDF and CBN@PVDF. The piezo-/ferroelectric field existing in CBN@PVDF mitigates the overpotential gradient at the interface and subsequently can yield a nearly uniform Zn deposit. As illustrated in Figure 4d for PVDF (and the corresponding current density shown in Figure S16a), there is an uneven distribution of Zn^2+^ concentration characterized by a pronounced concentration gradient at the dendrite tip. Its peak value is approximately five times higher than bulk Zn^2+^ concentrations. This localized increase in Zn^2+^ concentration exacerbates significant concentration polarization while contributing to heightened driving forces for dendrite formation.^57,58^ Under such strong concentration gradients coupled with low ionic conductivity inherent to SPEs, noncompact uneven morphologies are observed during Zn deposition (as evidenced by SEM images in Figure 4a), finally resulting in catastrophic battery failure. In contrast, both the Zn^2+^ concentration within CBN@PVDF with a piezo-/ferroelectric effect (Figure 4d) and the associated current density (Figure S16b) manifested a regular distribution. It is beneficial for alleviating the growth and spread of Zn dendrites. In conjunction with the simulation results depicted in Figure 4e, the abundant and homogeneous CBN within the SPEs can establish a robust and self-adjusting polarized electric field. This field alleviates excessive Zn^2+^ while refining heterogeneity in the deposition dynamics, thereby achieving overall uniformity in both Zn deposition and stripping.

### 2.4 Performances of Zn|CBN@PVDF|Pyrene-4,5,9,10-Tetraone (PTO) Batteries

After testing the excellent stability and reversibility of the Zn anode, full SSZIBs were assembled to evaluate compatibility between our fabricated CBN@PVDF alongside commercial PTO cathodes. First, linear sweep voltammetry (LSV) curves indicate that the CBN@PVDF exhibits an electrochemical stability window of 2.45 V (Figure S17), suggesting its ability to support the stable operation of the PTO cathodes in SSZIBs. As demonstrated by CV curves presented herein (Figure 5a), the two pairs of redox peaks in the CV curve of Zn|CBN@PVDF|PTO full cell were obtained at ≈1.21/0.99 and ≈0.76/0.55 V vs Zn/Zn^2+^ in the reduction and oxidation scans, respectively. The CV curves exhibit substantial overlap throughout subsequent cycles, proving the high stability of the Zn|CBN@PVDF|PTO battery. As presented by the GCD curves of Figure 5b, two consecutive discharge plateaus at 0.99 and 0.55 V coincide with the CV peaks at 0.2 C, yielding a high discharge capacity (295 mAh g^–1^). With an increasing current density, the GCD profiles retained two discharge plateaus along with superior gravimetric capacities. The Zn|CBN@PVDF|PTO full cell delivers decent specific capacities of 270, 248, 243, 230, and 221 mAh g^–1^ at 0.5, 1.0, 2.0, 5.0, and 10.0 C, respectively (Figure 5c). Then, we benchmark the rate performance of the Zn|CBN@PVDF|PTO full cells against other literature in Figure 5d to demonstrate the superiority of our fabricated CBN@PVDF among various SPEs.^47,48,52,59–64^ The exceptional rate performance of the solid Zn|CBN@PVDF|PTO full cell, capable of reaching up to 10 C, further substantiates the rapid Zn^2+^ migration kinetics facilitated by the CBN@PVDF. This remarkable enhancement can be attributed to its superior ionic conductivity and remarkable Zn^2+^ mobility, which are triggered by the outstanding dielectric properties of the CBN@PVDF.

**Figure 5:**
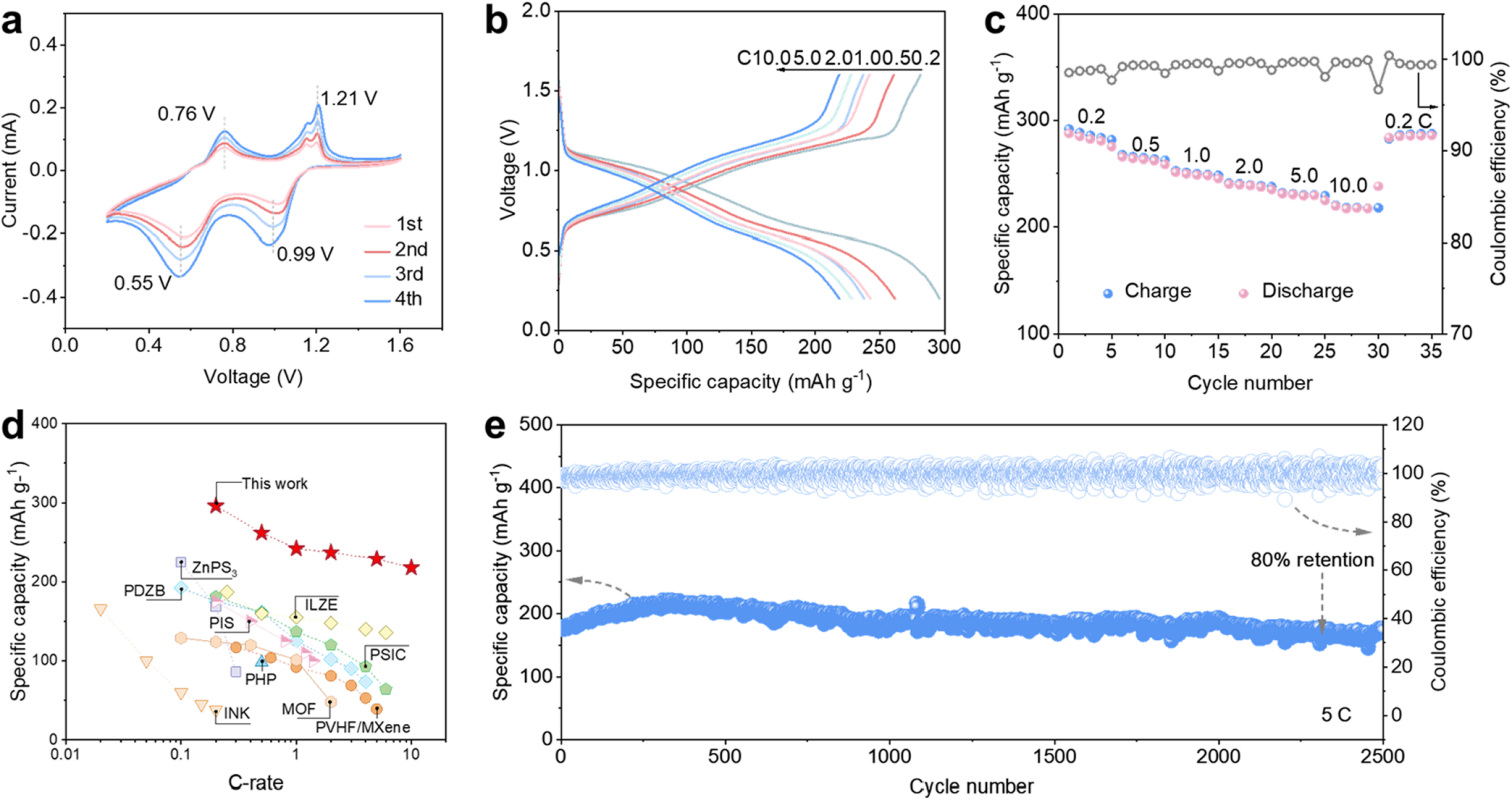
(a) CV curves of Zn|CBN@PVDF|PTO batteries measured at 0.2 mV s^–1^. The GCD curves (b) and rate performance (c) of Zn|CBN@PVDF|PTO cells at varied rates. (d) C-rate comparison of reported SSZIBs.^47,48,52,59–64^ (PHP: 2,6-bis­(propylimino)­methyl)-4-chlorophenol (Hbimcp) mixed with the poly­(propylene oxide) (PPO); PIS: polymers in salt; ILZE: ionic liquid (1-ethyl-3-methylimidazolium tetrafluoroborate ([EMIM]­BF_4_)) with a zinc salt (2m zinc tetrafluoroborate (Zn­(BF_4_)_2_)); PDZB: poly­(1,3-dioxolane, DOL)/zinc tetrafluoroborate (Zn­(BF_4_)_2_). (e) Cycle performance and CE of Zn|CBN@PVDF|PTO battery at 5C.

As illustrated in Figure 5e, long-term cycling tests are also carried out on Zn|CBN@PVDF|PTO solid cells. The durable Zn|CBN@PVDF|PTO batteries maintained an extended lifespan of 2500 cycles and exhibited impressive capacity retention of 80% even under a high discharge rate of 5 C. These exceptional characteristics were made possible by the piezo-/ferroelectric effect of CBN@PVDF, which effectively optimized the distribution of Zn^2+^ flux and controlled Zn deposition within the full cell. Furthermore, we employed commercial Na_2_Mn_0.6_Fe_0.2_Ni_0.2_[Fe­(CN)_6_] (PBA) cathodes as substitutes for the PTO cathode to verify the universality of the piezo-/ferroelectric effect enabled by CBN@PVDF. As presented in Figure S18a, the exceptional stability of the Zn|CBN@PVDF|PBA battery is evidenced by the well-overlapped CV curves. Additionally, this battery exhibits commendable rate performances (Figure S18b,c) and stably maintains a long 1000 cycle lifespan at 0.2 A g^–1^ (Figure S18d). To further explore the practical application potential of the fabricated CBN@PVDF, a solid Zn||tetra-chloro-benzoquinone (TCBQ) pouch cell (4 cm × 5 cm) based on CBN@PVDF with a high TCBQ loading mass (12.5 mg cm^–2^) was assembled and evaluated. As illustrated in Figure S19a,b, the pouch cell delivered a total capacity of 40 mAh and maintained a high-capacity retention (80%) after 300 cycles. This exceptional cycling stability stems from the rational design of CBN@PVDF SPEs. The piezoelectric and ferroelectric components within the SPE play critical roles in regulating ion transport. Specifically, they function by reducing the electrostatic potential difference and mitigating adverse ion aggregation within the EDL. Consequently, these mechanisms collectively suppress dendrite formation, while enabling remarkable capacity, excellent rate performance, and enhanced durability. Given this durable cycle performance, it can be concluded that incorporating functional SPEs with piezoelectric and ferroelectric components is a promising strategy for achieving high-performance SSZIBs suitable for wearable electronic devices.

## 3 Conclusion

We have introduced an innovative concept for fabricating multifunctional SPEs that incorporate a small amount of piezoelectric and ferroelectric phases. The assembled Zn|CBN@PVDF|Zn cells demonstrate remarkable deposition/stripping stability, cycling consistently for 2000 h at 0.5 mA cm^–2^ and 1500 h at 1.0 mA cm^–2^. The solid Zn|CBN@PVDF|PTO cell exhibited an outstanding rate performance, reaching up to 10 C due to the high dielectricity of CBN@PVDF. It also delivered a long lifespan of 2500 cycles, maintaining 80% capacity retention at a high rate of 5 C. Our findings indicate that the polarization field effectively regulates Zn deposition, as simulation and experimental results confirm. COMSOL simulations reveal that the piezo-/ferroelectric field formed during operation actively counteracts the driving force for dendrite formation at electrode–electrolyte interfaces, optimizes Zn^2+^ flux distribution, and promotes uniform Zn deposition. These outcomes highlight significant implications regarding the utilization of multimodal materials aimed at enhancing the functional performance of SSZIBs. This innovative strategy presents promising opportunities for achieving dendrite-free cycling, which is highly relevant for SSZIBs and extends to broader electrochemical applications.

## Supplementary Material



## Notes

Complete experimental procedures, details of the computational methods, additional material characterization, and electrochemical studies of the solid electrolytes and batteries (PDF)

## References

[1] Liu C., Xu W., Mei C., Li M., Chen W., Hong S., Kim W. Y., Lee Sy., Wu Q. (2021). A Chemically Self-Charging
Flexible Solid-State Zinc-Ion Battery Based on VO_2_ Cathode
and Polyacrylamide–Chitin Nanofiber Hydrogel Electrolyte. Adv. Energy Mater..

[2] Man Q., An Y., Shen H., Wei C., Zhang X., Wang Z., Xiong S., Feng J. (2023). MXenes and
Their Derivatives for
Advanced Solid-State Energy Storage Devices. Adv. Funct. Mater..

[3] Tu W. B., Liang S., Song L. N., Wang X. X., Ji G. J., Xu J. J. (2024). Nanoengineered Functional
Cellulose Ionic Conductor Toward High-
Performance All-Solid-State Zinc-Ion Battery. Adv. Funct. Mater..

[4] Li C., Wu Q., Ma J., Pan H., Liu Y., Lu Y. (2022). Regulating
zinc metal anodesvianovel electrolytes in rechargeable zinc-based
batteries. J. Mater. Chem. A.

[5] Liu C., Xie X., Lu B., Zhou J., Liang S. (2021). Electrolyte Strategies
toward Better Zinc-Ion Batteries. ACS Energy
Lett..

[6] Yi Z., Chen G., Hou F., Wang L., Liang J. (2020). Strategies
for the Stabilization of Zn Metal Anodes for Zn-Ion Batteries. Adv. Energy Mater..

[7] Tian Y., Chen S., He Y., Chen Q., Zhang L., Zhang J. (2022). A highly reversible
dendrite-free Zn anode via spontaneous galvanic
replacement reaction for advanced zinc-iodine batteries. Nano Res. Energy.

[8] Li Z., Huang J., Liaw B. Y., Metzler V., Zhang J. (2014). A review of
lithium deposition in lithium-ion and lithium metal secondary batteries. J. Power Sources.

[9] Luo S., Wang Z., Li X., Liu X., Wang H., Ma W., Zhang L., Zhu L., Zhang X. (2021). Growth of lithium-indium
dendrites in all-solid-state lithium-based batteries with sulfide
electrolytes. Nat. Commun..

[10] Wu J. (2022). Understanding
the Electric Double-Layer Structure, Capacitance, and Charging Dynamics. Chem. Rev..

[11] Zheng J., Archer L. A. (2021). Controlling electrochemical growth
of metallic zinc
electrodes: Toward affordable rechargeable energy storage systems. Sci. Adv..

[12] Yang Q., Li Q., Liu Z., Wang D., Guo Y., Li X., Tang Y., Li H., Dong B., Zhi C. (2020). Dendrites
in Zn-Based Batteries. Adv. Mater..

[13] Zou P., Zhang R., Yao L., Qin J., Kisslinger K., Zhuang H., Xin H. L. (2021). Ultrahigh-Rate and Long-Life Zinc–Metal
Anodes Enabled by Self-Accelerated Cation Migration. Adv. Energy Mater..

[14] Zhao Q., Stalin S., Zhao C.-Z., Archer L. A. (2020). Designing solid-state
electrolytes for safe, energy-dense batteries. Nat. Rev. Mater..

[15] Xu Y., Dong K., Jie Y., Adelhelm P., Chen Y., Xu L., Yu P., Kim J., Kochovski Z., Yu Z., Li W., LeBeau J., Shao-Horn Y., Cao R., Jiao S., Cheng T., Manke I., Lu Y. (2022). Promoting
Mechanistic Understanding of Lithium Deposition and Solid-Electrolyte
Interphase (SEI) Formation Using Advanced Characterization and Simulation
Methods: Recent Progress, Limitations, and Future Perspectives. Adv. Energy Mater..

[16] Zhang K. L., Li N., Li X., Huang J., Chen H., Jiao S., Song W. L. (2022). Understanding
Enhanced Ionic Conductivity in Composite
Solid-State Electrolyte in a Wide Frequency Range of 10(−2)
−10(10) Hz. Adv. Sci..

[17] Fu J., Yang S., Hou J., Azhari L., Yao Z., Ma X., Liu Y., Vanaphuti P., Meng Z., Yang Z., Zhong Y., Wang Y. (2023). Modeling assisted synthesis of Zr-doped
Li3-xIn1-xZrxCl6 with ultrahigh ionic conductivity for lithium-ion
batteries. J. Power Sources.

[18] Kondo H., Sawada H., Okuda C., Sasaki T. (2019). Influence of the Active
Material on the Electronic Conductivity of the Positive Electrode
in Lithium-Ion Batteries. J. Electrochem. Soc..

[19] Siroma Z., Sato T., Takeuchi T., Nagai R., Ota A., Ioroi T. (2016). AC impedance analysis
of ionic and electronic conductivities in electrode
mixture layers for an all-solid-state lithium-ion battery. J. Power Sources.

[20] Chen H., Adekoya D., Hencz L., Ma J., Chen S., Yan C., Zhao H., Cui G., Zhang S. (2020). Stable Seamless Interfaces
and Rapid Ionic Conductivity of Ca–CeO2/LiTFSI/PEO Composite
Electrolyte for High-Rate and High-Voltage All-Solid-State Battery. Adv. Energy Mater..

[21] Li N., Luo J., Zhu J., Zhuang X. (2023). Cathodic interface in sulfide-based
all-solid-state lithium batteries. Energy Storage
Mater..

[22] Zhang Z., Wang P., Wei C., Feng J., Xiong S., Xi B. (2024). Synchronous Regulation
of D-Band Centers in Zn Substrates and Weakening
Pauli Repulsion of Zn Ions Using the Ascorbic Acid Additive for Reversible
Zinc Anodes. Angew. Chem., Int. Ed..

[23] Wang X., Ying Y., Li X., Chen S., Gao G., Huang H., Ma L. (2023). Preferred planar crystal growth and
uniform solid electrolyte interfaces enabled by anion receptors for
stable aqueous Zn batteries. Energy Environ.
Sci..

[24] Yang Y., Yang H., Zhu R., Zhou H. (2023). High reversibility
at high current density: the zinc electrodeposition principle behind
the “trick. Energy Environ. Sci..

[25] Ge H., Feng X., Liu D., Zhang Y. (2023). Recent advances and
perspectives for Zn-based batteries: Zn anode and electrolyte. Nano Res. Energy.

[26] Guo X., Ju Z., Qian X., Liu Y., Xu X., Yu G. (2023). A Stable Solid
Polymer Electrolyte for Lithium Metal Battery with Electronically
Conductive Fillers. Angew. Chem., Int. Ed..

[27] Braga M. H., Oliveira J. E., Murchison A. J., Goodenough J. B. (2020). Performance
of a ferroelectric glass electrolyte in a self-charging electrochemical
cell with negative capacitance and resistance. Appl. Phys. Rev..

[28] Swift M. W., Qi Y. (2019). First-Principles Prediction
of Potentials and Space-Charge Layers
in All-Solid-State Batteries. Phys. Rev. Lett..

[29] Swift M. W., Swift J. W., Qi Y. (2021). Modeling the
electrical double layer
at solid-state electrochemical interfaces. Nat.
Comput. Sci..

[30] Wang L., Xie R., Chen B., Yu X., Ma J., Li C., Hu Z., Sun X., Xu C., Dong S., Chan T.-S., Luo J., Cui G., Chen L. (2020). In-situ visualization of the space-charge-layer
effect on interfacial lithium-ion transport in all-solid-state batteries. Nat. Commun..

[31] Pei Z. (2022). Symmetric
is nonidentical: Operation history matters for Zn metal anode. Nano Res. Energy.

[32] Liao B., Liao X., Xie H., Qin Y., Zhu Y., Yu Y., Hou S., Zhang Y., Fan X. (2022). Built in electric
field
boosted photocatalytic performance in a ferroelectric layered material
SrBi2Ta2O9 with oriented facets: Charge separation and mechanism insights. J. Mater. Sci. Technol..

[33] Zhang Y., Jie W., Chen P., Liu W., Hao J. (2018). Ferroelectric and Piezoelectric
Effects on the Optical Process in Advanced Materials and Devices. Adv. Mater..

[34] Chen H., Zhai J. (2012). Enhancing Piezoelectric
Performance of CaBi2Nb2O9 Ceramics Through
Microstructure Control. J. Electron. Mater..

[35] Liu Q., Zhan F., Luo H., Luo X., Yi Q., Sun Q., Xiao Z., Zhang Y., Zhang D., Bowen C. R. (2023). Na-Sm Bimetallic
Regulation and Band Structure Engineering in CaBi2Nb2O9 to Enhance
Piezo-photo-catalytic Performance. Adv. Funct.
Mater..

[36] Chen M., Wang Y., Niu Y., Chen X., Su H., Xia L., Liu C., Zhou J., Wang Z., Li B., Lu D. (2026). PH-Responsive CaCO3 Nanoplatform Amplifies SDT via
Calcium Overload-ROS
Loop for Deep Tumor Therapy. iScience.

[37] Alharbi S. R., Alhassan M., Jalled O., Wageh S., Saeed A. (2018). Structural
characterizations and electrical conduction mechanism of CaBi2Nb2O9
single-phase nanocrystallites synthesized via sucrose-assisted sol–gel
combustion method. J. Mater. Sci..

[38] Aswin G., Prakash V., Subasree N., Arul V., Paramasivam P., Gnanasekaran L., Santhamoorthy M., Radhakrishnan K. (2026). Recent advances
in corrosion behaviour and interfacial failure of rubber–metal
composites. npj Mater. Degrad..

[39] Joshi S., Acharya S., Sayyad S., Shirbhate S., Quazi T., Dorle N. (2025). Enhancing the β-phase of PVDF
by nano piezoceramic hybrid for advanced capacitive and energy storage
application. J. Electroceram..

[40] Liu Z., Lei Y., Zhang X., Kang Z., Zhang J. (2022). Effect Mechanism and
Simulation of Voids on Hygrothermal Performances of Composites. Polymers.

[41] Wu Y., Li Y., Wang Y., Liu Q., Chen Q., Chen M. (2022). Advances and
prospects of PVDF based polymer electrolytes. J. Energy Chem..

[42] Hou Q., Yang B., Ma C., Zhou Z., Liang R., Li H., Dong X. (2022). Tailoring structure and piezoelectric properties of
CaBi2Nb2O9 ceramics by W6+-Doping. Ceram. Int..

[43] Jiang Z., Tan X., Xu J., Huang Y. (2022). Piezoelectric-Induced Internal Electric
Field in Bi2WO6 Nanoplates for Boosting the Photocatalytic Degradation
of Organic Pollutants. ACS Appl. Nano Mater..

[44] Lin E., Qin N., Wu J., Yuan B., Kang Z., Bao D. (2020). BaTiO­(3) Nanosheets
and Caps Grown on TiO(2) Nanorod Arrays as Thin-Film Catalysts for
Piezocatalytic Applications. ACS Appl. Mater.
Int..

[45] Duan H., Yin Y. X., Shi Y., Wang P. F., Zhang X. D., Yang C. P., Shi J. L., Wen R., Guo Y. G., Wan L. J. (2018). Dendrite-Free Li-Metal Battery Enabled by a Thin Asymmetric
Solid Electrolyte with Engineered Layers. J.
Am. Chem. Soc..

[46] Zhang X., Wang S., Xue C., Xin C., Lin Y., Shen Y., Li L., Nan C. W. (2019). Self-Suppression
of Lithium Dendrite in All-Solid-State Lithium Metal Batteries with
Poly­(vinylidene difluoride)-Based Solid Electrolytes. Adv. Mater..

[47] Chen Z., Li X., Wang D., Yang Q., Ma L., Huang Z., Liang G., Chen A., Guo Y., Dong B., Huang X., Yang C., Zhi C. (2021). Grafted MXene/polymer
electrolyte for high performance solid zinc batteries with enhanced
shelf life at low/high temperatures. Energy
Environ. Sci..

[48] Chen Z., Wang T., Hou Y., Wang Y., Huang Z., Cui H., Fan J., Pei Z., Zhi C. (2022). Polymeric Single-Ion
Conductors with Enhanced Side-Chain Motion for High-Performance Solid
Zinc-Ion Batteries. Adv. Mater..

[49] Li Y., Yang X., He Y., Li F., Ouyang K., Ma D., Feng J., Huang J., Zhao J., Yang M., Wang Y., Xie Y., Mi H., Zhang P. (2023). A Novel Ultrathin
Multiple-Kinetics-Enhanced Polymer Electrolyte Editing Enabled Wide-Temperature
Fast-Charging Solid-State Zinc Metal Batteries. Adv. Funct. Mater..

[50] Liu J., Khanam Z., Muchakayala R., Song S. (2020). Fabrication and characterization
of Zn-ion-conducting solid polymer electrolyte films based on PVdF-HFP/Zn­(Tf)­2
complex system. J. Mater. Sci..

[51] Wang Z., Chen H., Wang H., Huang W., Li H., Pan F. (2022). In Situ Growth of a
Metal–Organic Framework-Based Solid Electrolyte
Interphase for Highly Reversible Zn Anodes. ACS Energy Lett..

[52] Wang Z., Hu J., Han L., Wang Z., Wang H., Zhao Q., Liu J., Pan F. (2019). A MOF-based
single-ion Zn2+ solid electrolyte leading
to dendrite-free rechargeable Zn batteries. Nano Energy.

[53] Yang H., Huq R., Farrington G. (1990). Conductivity in PEO-based Zn­(II) polymer
electrolytes. Solid State Ionics.

[54] Tian C., Wang J., Sun R., Ali T., Wang H., Xie B. B., Zhong Y., Hu Y. (2023). Improved Interfacial
Ion Migration and Deposition through the Chain-Liquid Synergistic
Effect by a Carboxylated Hydrogel Electrolyte for Stable Zinc Metal
Anodes. Angew. Chem., Int. Ed..

[55] Qiu H., Du X., Zhao J., Wang Y., Ju J., Chen Z., Hu Z., Yan D., Zhou X., Cui G. (2019). Zinc anode-compatible
in-situ solid electrolyte interphase via cation solvation modulation. Nat. Commun..

[56] Li Q., Chen A., Wang D., Pei Z., Zhi C. (2022). Soft Shorts”
Hidden in Zinc Metal Anode Research. Joule.

[57] Cui B.-F., Han X.-P., Hu W.-B. (2021). Micronanostructured
Design of Dendrite-Free
Zinc Anodes and Their Applications in Aqueous Zinc-Based Rechargeable
Batteries. Small Struct..

[58] Chen L., Zhang H. W., Liang L. Y., Liu Z., Qi Y., Lu P., Chen J., Chen L.-Q. (2015). Modulation of dendritic patterns
during electrodeposition: A nonlinear phase-field model. J. Power Sources.

[59] Bu F., Li C., Wang Q., Liu X. (2022). Ultraviolet-assisted printing of
flexible all-solid-state zinc batteries with enhanced interfacial
bond. Chem. Eng. J..

[60] Liu D., Tang Z., Luo L., Yang W., Liu Y., Shen Z., Fan X. H. (2021). Self-Healing
Solid Polymer Electrolyte
with High Ion Conductivity and Super Stretchability for All-Solid
Zinc-Ion Batteries. ACS Appl. Mater. Int..

[61] Lv Z., Kang Y., Chen G., Yang J., Chen M., Lin P., Wu Q., Zhang M., Zhao J., Yang Y. (2024). Stable Solid-State
Zinc–Iodine Batteries Enabled by an Inorganic ZnPS3 Solid Electrolyte
with Interconnected Zn2+ Migration Channels. Adv. Funct. Mater..

[62] Ma L., Chen S., Li N., Liu Z., Tang Z., Zapien J. A., Chen S., Fan J., Zhi C. (2020). Hydrogen-Free
and Dendrite-Free All-Solid-State Zn-Ion Batteries. Adv. Mater..

[63] Ma L., Chen S., Li X., Chen A., Dong B., Zhi C. (2020). Liquid-Free All-Solid-State
Zinc Batteries and Encapsulation-Free
Flexible Batteries Enabled by In Situ Constructed Polymer Electrolyte. Angew. Chem., Int. Ed..

[64] Yan K., Fan Y., Hu F., Li G., Yang X., Wang X., Li X., Peng C., Wang W., Fan H., Ma L. (2024). A “Polymer-in-Salt”
Solid Electrolyte Enabled by Fast Phase Transition Route for Stable
Zn Batteries. Adv. Funct. Mater..

