# Supplementary material for: Leveraging Piezoelectric and Ferroelectric Effects to Control Zinc Deposition for High-Performance Solid-State Zinc Batteries
Source: J Am Chem Soc. 2026 Apr 16;148(16):16872–81. doi: 10.1021/jacs.5c23299 (PMC13133918; doi:10.1021/jacs.5c23299)
Supplement: Supplementary file 1 [file ja5c23299_si_001.pdf]

Leveraging Piezoelectric and Ferroelectric Effects to Control Zinc Deposition for High-Performance Solid-State Zinc Batteries

Yue Hou[1,∇], Qiong Liu[4,∇], Zeru Wang[2], Xinru Yang[3], Dedi Li[3], Yiqiao Wang[3], Zhiquan Wei[3], Zhaodong Huang[9], Qing Li[5], Ke Wang[2], Chunyi Zhi[1,3,6,7,8]**

[1]Hong Kong Center for Cerebro-Cardiovascular Health Engineering (COCHE), Shatin, New Territories, Hong Kong 999077, China

[2]School of System Design and Intelligent Manufacturing, Southern University of Science and Technology, Shenzhen 518055, China

[3]Department of Materials Science and Engineering, City University of Hong Kong, 83 Tat Chee Avenue, Kowloon, Hong Kong 999077, China

[4]College of Physics, Sichuan University, Chengdu, Sichuan 610065, China

[5]Institute of Applied Physics and Materials Engineering, University of Macau, Macau 999078, China

[6]Department of Mechanical Engineering, The University of Hong Kong, Pokfulam, Hong Kong 999077, China

[7]Materials Innovation Institute for Life Sciences and Energy (MILES), HKU-SIRI, Shenzhen 518048, China

[8]Center for Energy Storage, The University of Hong Kong, Pokfulam, Hong Kong 999077, China

[9]Department of Chemical and Biological Engineering, Hong Kong University of Science and Technology, Clear Water Bay, Kowloon, Hong Kong 999077, China

*E-mail: wangk7@sustech. edu. cn; cy.zhi@cityu.edu.hk

Experimental and Methodology

1. Materials preparation

N-Methyl-2-pyrrolidone (NMP), N, N-Dimethylformamide (DMF), polyvinylidene difluoride (PVDF), Calcium carbonate ($CaCO_3$), Bismuth Trioxide (Bi_2O_3), Niobium pentoxide (Nb_2O_5), Sodium chloride (NaCl), Potassium chloride (KCl), Zinc trifluromethanesulfonate ($Zn(OTf)_2$), pyrene-4,5,9,10-tetraone (PTO), $Na_2Mn_{0.6}Fe_{0.2}Ni_{0.2}[Fe(CN)_6]$(PBA), tetra-chloro-benzoquinone (TCBQ) are all purchased from Aladdin.

2. Synthesis of $CaBi_2Nb_2O_9$ (CBN) sheets

A traditional molten-salt growth method was employed to fabricate CBN sheets. The synthesis process involved mixing raw materials in ethanol based on their stoichiometric ratios, including $CaCO_3$ (3.61 g), Bi_2O_3 (17.69 g), and Nb_2O_5 (10.09 g), along with NaCl (13.19 g) and KCl (16.81 g). The combined materials underwent ball grinding for 24 hours at 300 rotations per minute, followed by drying in an oven and calcination at 950 °C for 6 hours at a heating rate of 5 °C per minute. Subsequently, deionized water was utilized to wash the powder thoroughly, removing the molten salt residues of NaCl and KCl from the synthesized material.

3. Preparation of PVDF membrane

0.5g of PVDF polymer was added into a beaker containing 5 mL DMF. Subsequently, $Zn(OTf)_2$ (0.5 g) was dissolved in the DMF solution. The solution was stirred for 2 hours and heated at 70°C. The resulting transparent solution was carefully dripped onto a glass plate. The coated plate was then dried in an oven at 50°C for 8 hours to eliminate residual DMF.

4. Preparation of CBN@PVDF membrane

0.5 g of PVDF polymer was placed into a beaker with 5 mL of DMF. Subsequently, $Zn(OTf)_2$ (0.5 g) and CBN platelets (0.025 g) were dissolved in the DMF solution. The mixture was then stirred for 2 hours and heated at 70 °C. Afterward, the resulting transparent solution was carefully deposited onto a glass plate. The coated plate was then dried in an oven at 50°C for 8 hours to eliminate any remaining DMF, ensuring a uniform film comprising the PVDF polymer, $Zn(OTf)_2$, and CBN platelets formed.

5. Preparation for PTO cathode

The cathode was prepared by coating PTO powder onto carbon cloth with a loading mass of 2 mg cm^{-2} (PTO: PVDF: carbon black = 8:1:1).

6. Preparation for PBA cathode

The cathode was prepared by coating PBA powder onto carbon cloth with a loading mass of 2 mg cm^{-2} (PBA: PVDF: carbon black = 8:1:1).

7. Preparation for TCBQ cathode

The cathode was prepared by coating TCBQ powder onto Ti mesh with a loading mass of 12.5 mg cm^{-2} (TCBQ: PVDF: carbon black = 8:1:1).

8. Characterization methods

Fourier-transform infrared spectroscopy (FTIR) was performed using the PerkinElmer Spectrum II. Raman spectra were conducted on the WITec alpha300 R Raman System. Differential scanning calorimetry (DSC) experiments were carried out using a DSC Q2000 (TA Instruments) with Tzero pans. Morphological investigations were done using the field-emission scanning electron microscope (SEM, FEI Quanta 450 FEG) and the high-resolution transmission electron microscopy (HRTEM, JEOL-2001F). Powder X-ray diffraction (PXRD) was performed at ambient temperature on

Bruker D2 Phaser equipment, using Cu-Kα radiation with a monochromator. The discharged/charged Zn anodes were gently washed with ethanol three times and then dried at room temperature for 24 h before measurements. Piezo-response force microscopy (PFM, Bruker Multimode8) was utilized to analyze the piezo-response of CBN and CBN@PVDF. To investigate potential changes at the interface between the solid polymer electrolyte (SPE) and the Zn anode, Kelvin probe force microscopy (KPFM) in the atomic force microscopy (AFM) technique was employed. The AFM-nano-infrared examinations were conducted using the Bruker Anasys nanoIR2-fs instrument to characterize the materials and their properties further.

9. Electrochemical measurement

To calculate the ionic conductivity of SPE based on the data obtained from the electrochemical impedance spectroscopy (EIS) measurements, you can use the following equation:

$$\sigma=L/(A*R)$$

where L is the thickness of SPE, R is the bulk resistance of SPE, and S is the area of Ti metal.

The Zn||Zn symmetric batteries with our fabricated SPE were conducted EIS measurements from 10 MHz to 0.01 Hz with a 20 mV AC oscillation. The transference number of cations, including Zn^{2+} can be calculated according to the following equation:

$$t_{Li^+} = \frac{I_S(\Delta V - I_0 R_0)}{I_0(\Delta V - I_S R_S)}$$

where I_s and I_0 are the steady-state and initial currents, respectively, R_0 and R_s are the interfacial resistance before and after polarization.

The electrochemical stability window of the SPE was assessed by performing

linear sweep voltammetry (LSV) measurements on the assembled Zn|SPE|Ti cells. All these electrochemical properties were carried out on the CHI 760D electrochemical workstation. CR2032 coin cells were constructed for electrochemical measurements employing a Zn foil as the anode, PTO or PBA as the cathode, and PVDF or CBN@PVDF as solid electrolytes. The cycling tests were conducted using the Land CT2001A battery testing system.

10. COMSOL calculations

The simulations were conducted in COMSOL Multiphysics 6.0 using partial differential equations in a 2D geometry. A two-dimensional mesh of 100 × 100 grids was employed, with each grid representing 1 μm. An adaptive time model with a time step of 0.2 s was utilized, and the simulation was set to run for a maximum time of 600 seconds. The specific parameters employed in this phase-field model are detailed in **Table S1**.

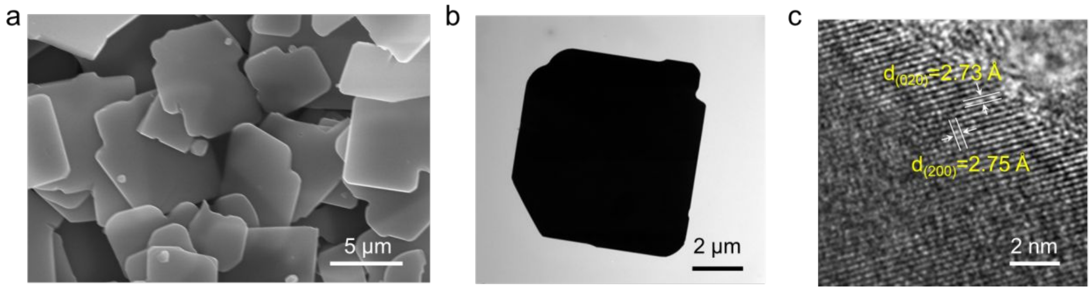

Figure S1. SEM image (a) and HRTEM image (b, c) of the fabricated CBN.

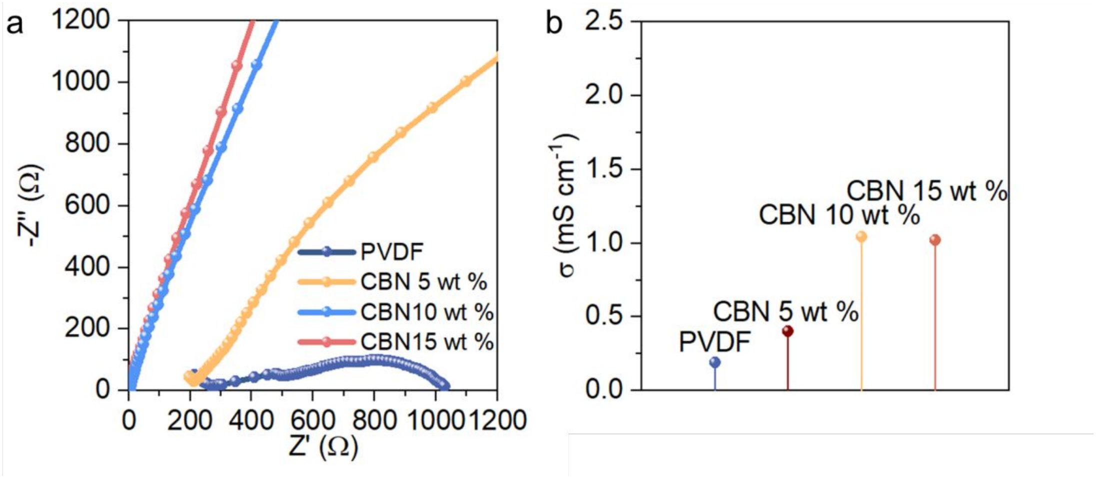

Figure S2. The EIS patterns (a) and the corresponding ionic conductivities (b) of the fabricated PVDF composites with different CBN contents.

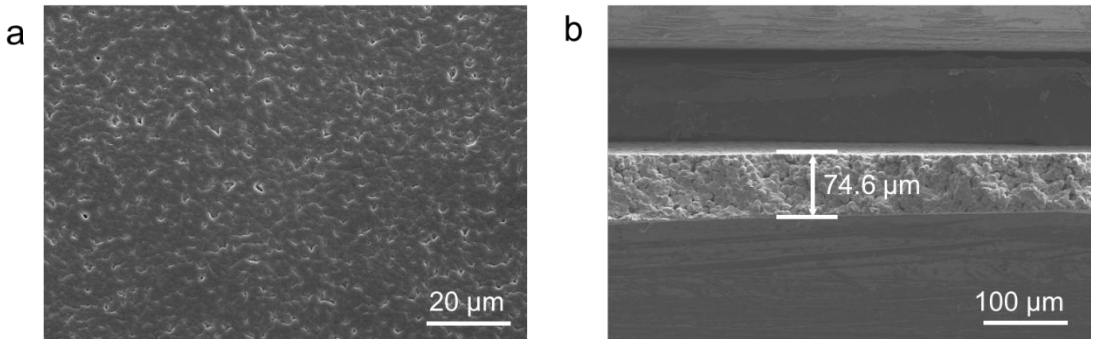

Figure S3. (a) The SEM image and (b) the cross-sectional SEM image of the fabricated CBN@PVDF.

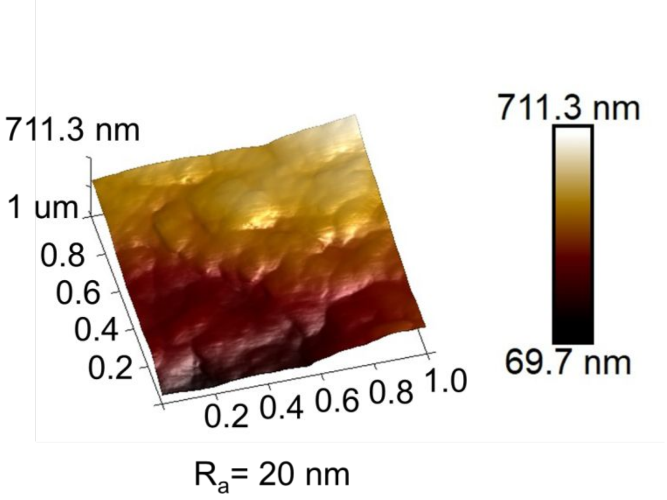

Figure S4. The AFM image of the fabricated CBN@PVDF.

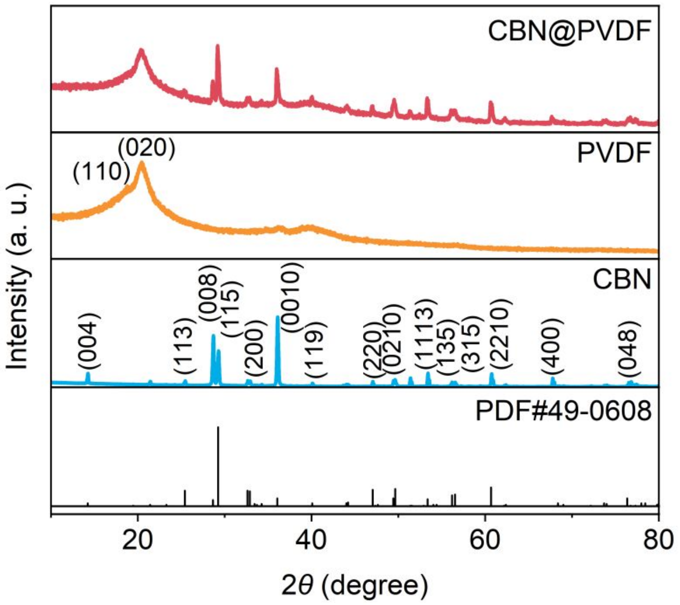

Figure S5. The XRD patterns of the fabricated CBN, PVDF, and CBN@PVDF.

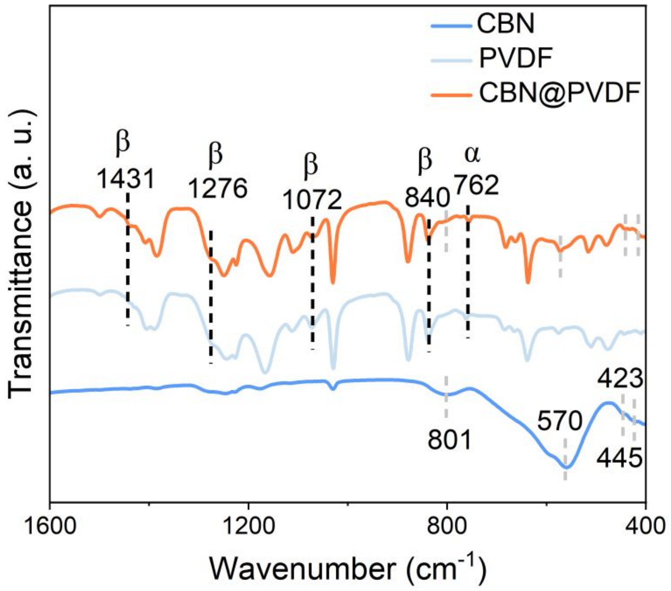

Figure S6. The FTIR curves of the fabricated CBN, PVDF and CBN@PVDF.

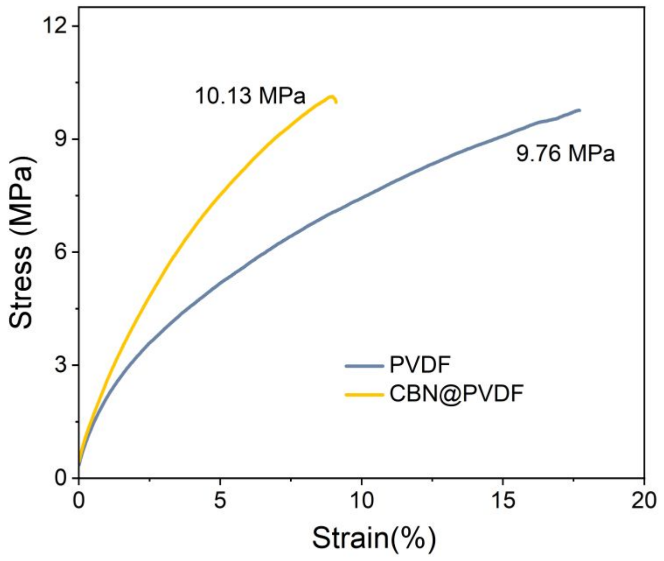

Figure S7. The stress-strain curves of the fabricated PVDF and CBN@PVDF.

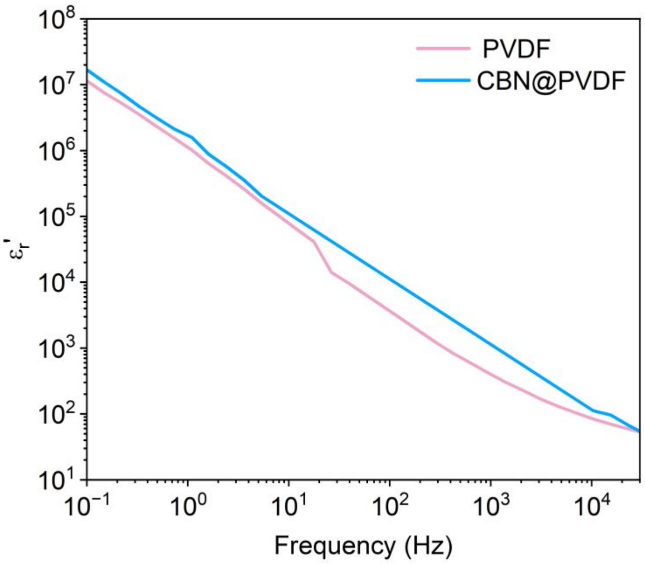

Figure S8. Real part (ε_r') of relative permittivity as a function of temperature at different frequencies for CBN@PVDF and PVDF.

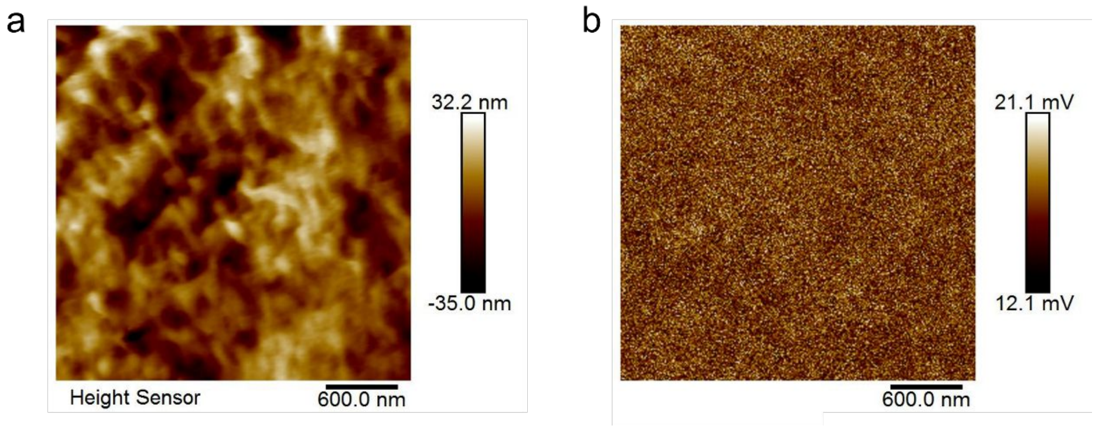

Figure S9. The PFM (a) height image and (b) amplitude image of the fabricated CBN@PVDF at a bias of -0.5 V.

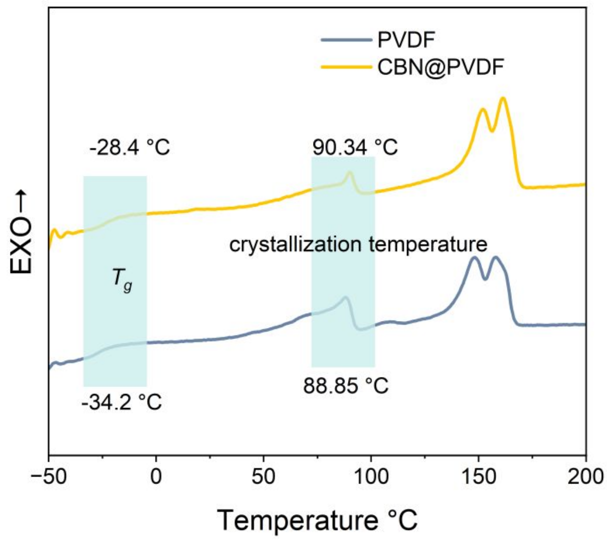

Figure S10. DSC curves of the fabricated PVDF and CBN@PVDF.

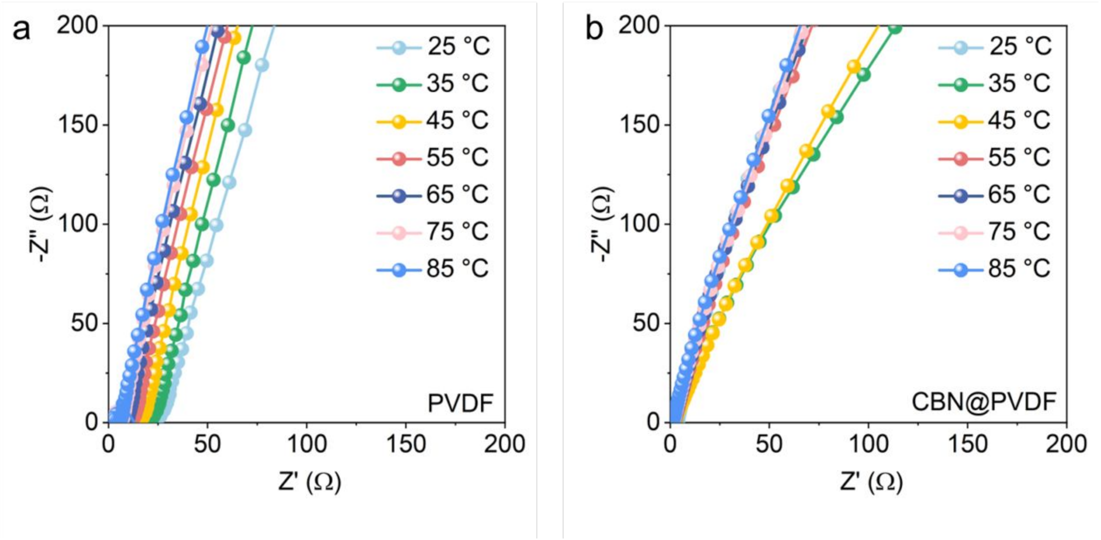

Figure S11. The EIS patterns of the PVDF (a) and CBN@PVDF (b) in a broad temperature range of 25 to 85 °C.

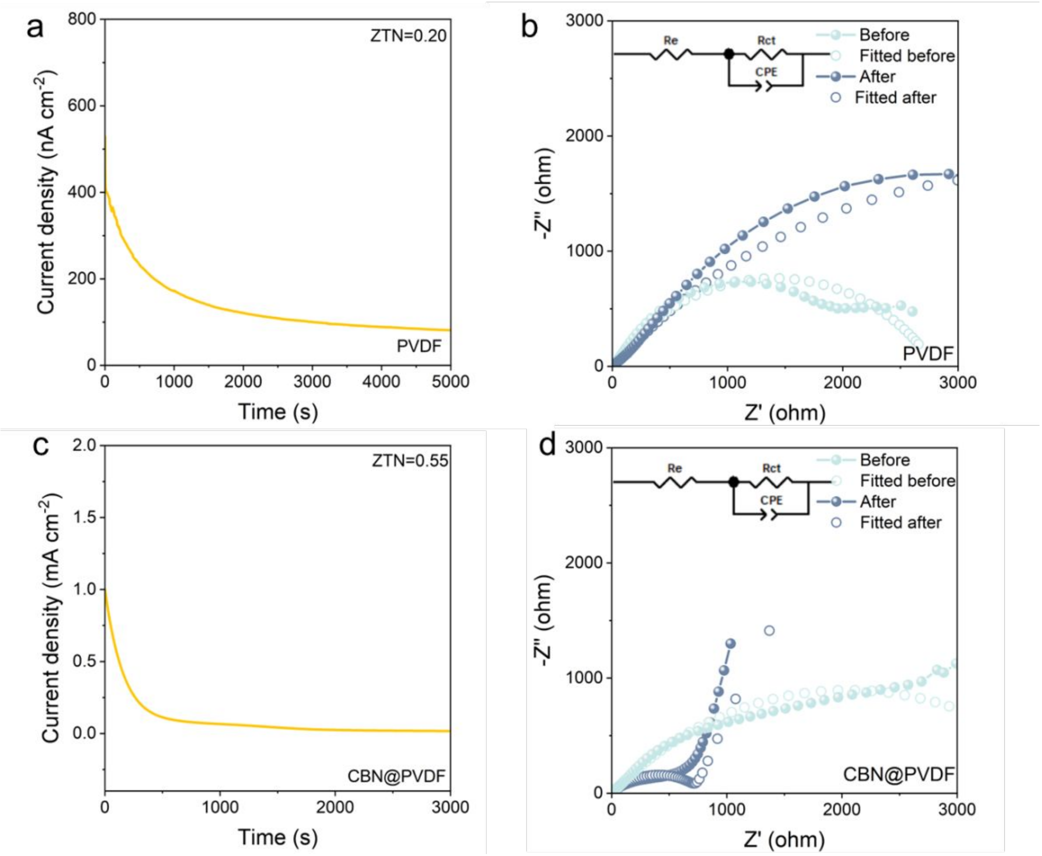

Figure S12. Chronoamperometry profiles of the Zn|PVDF|Zn under a polarization voltage of 20 mV (a) and the corresponding EIS patterns (b) before and after Chronoamperometry tests. Chronoamperometry profiles of the Zn|CBN@PVDF|Zn under a polarization voltage of 20 mV (c) and the corresponding EIS patterns (d) before and after Chronoamperometry tests.

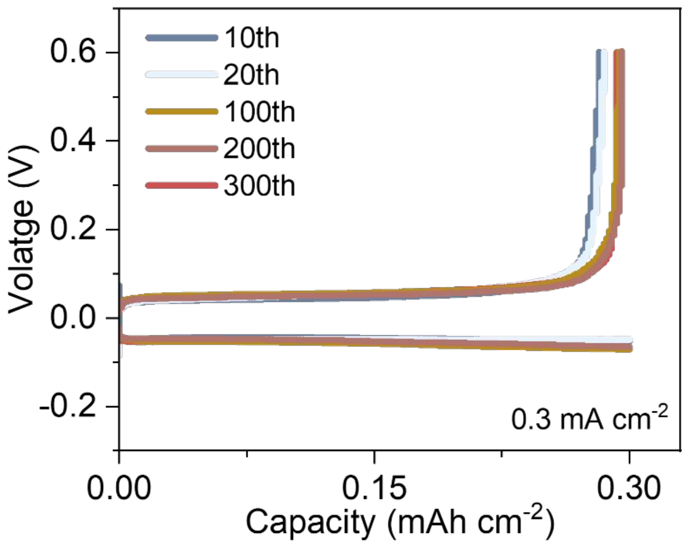

Figure S13. GCD curves of the Zn||Ti cells assembled by CBN@PVDF.

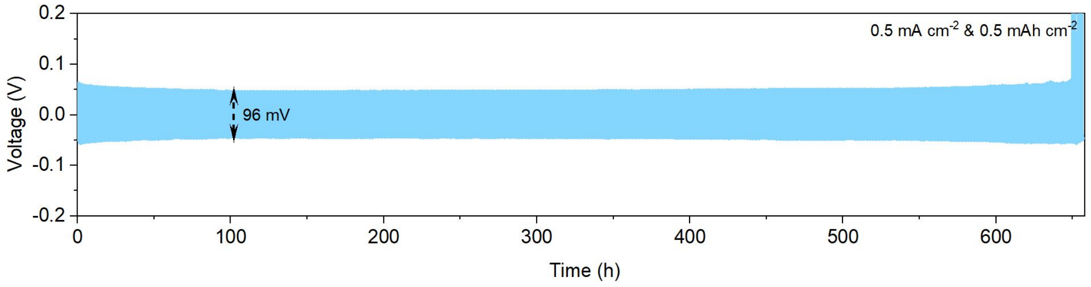

Figure S14. Long-term cycling test of the Zn|PVDF|Zn cells at 0.5 mA cm^{-2} with 0.5 mAh cm^{-2}.

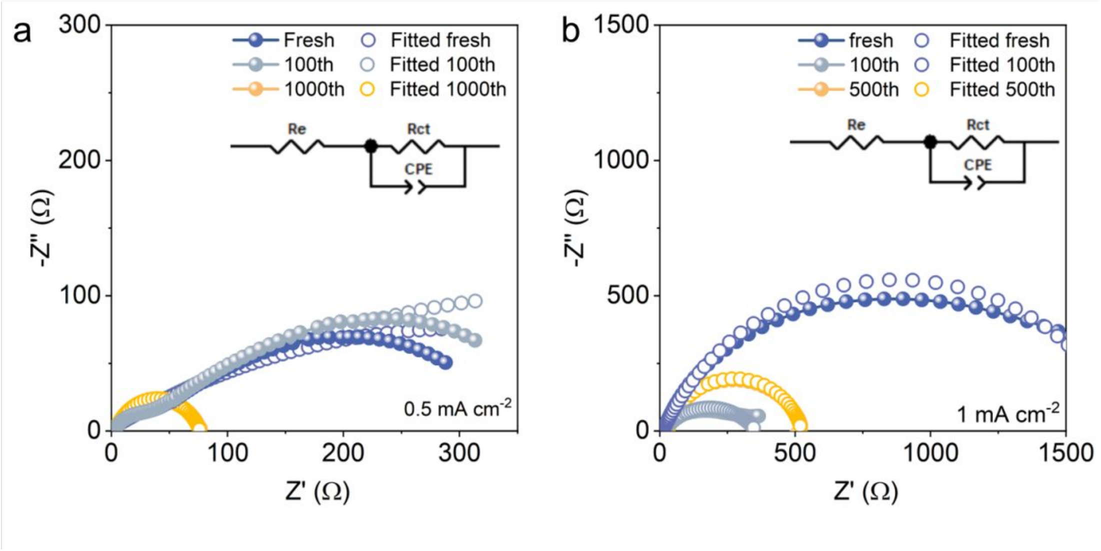

Figure S15. EIS patterns at different charge conditions during the long-term cycling test of the Zn|CBN@PVDF|Zn cells at 0.5 mA cm^{-2} (a) and 1.0 mA cm^{-2} (b).

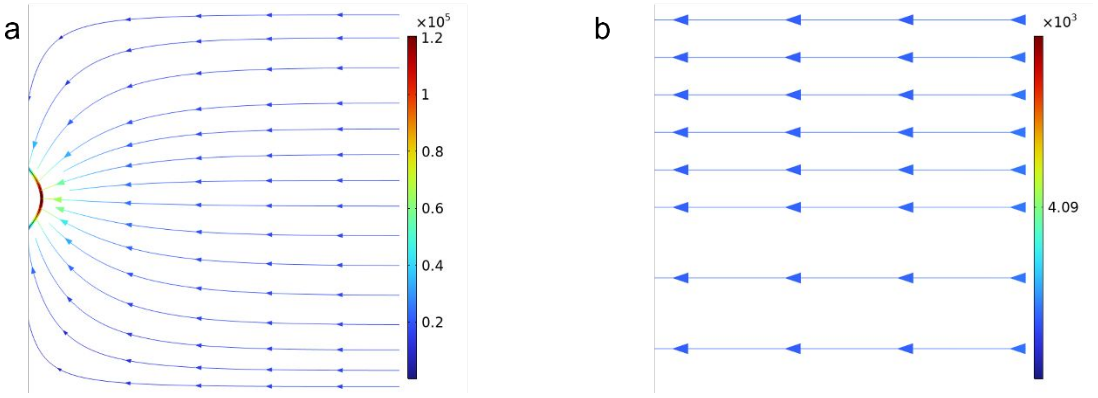

Figure S16. The current density distribution for the PVDF (a) and CBN@PVDF (b) after 600 s of 1 mA cm^{-2}.

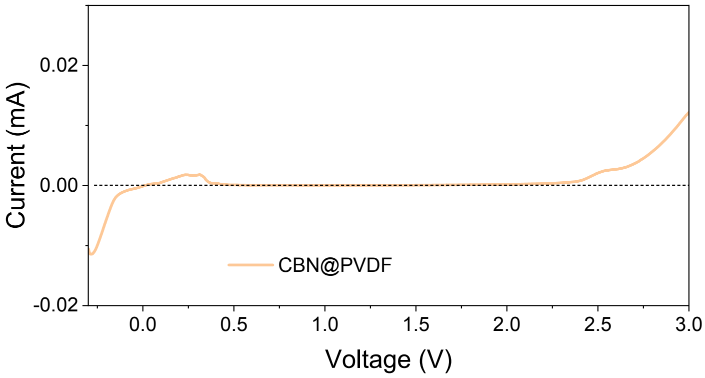

Figure S17. LSV curves of Zn|CBN@PVDF|Ti cells at 0.5 mV s^{-1}.

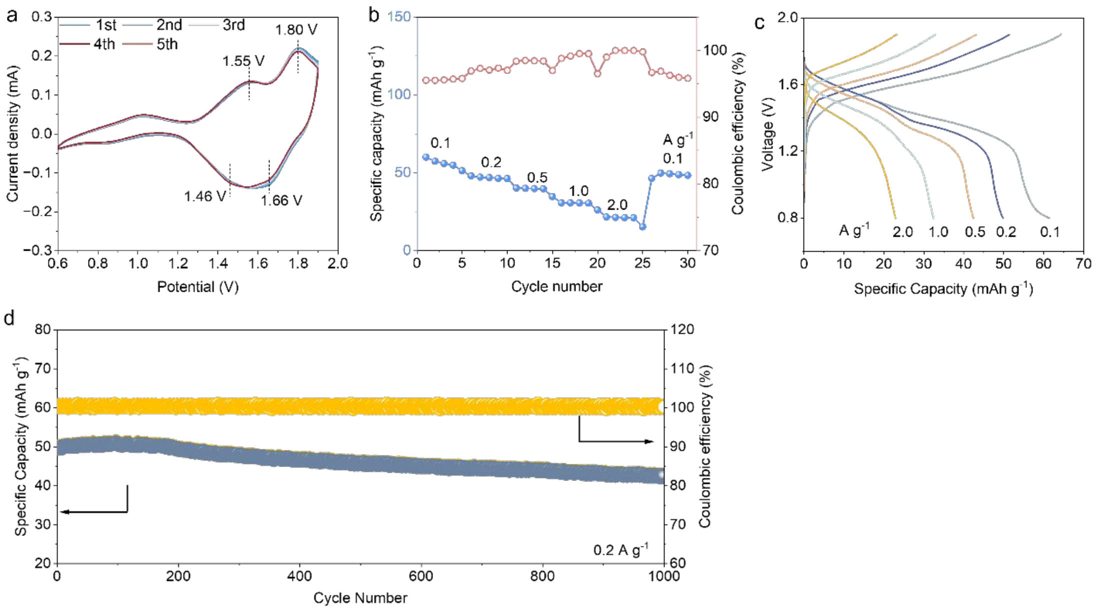

Figure S18. (a) CV curves of Zn|CBN@PVDF|PBA cells at 0.5 mV s^{-1}. (b) Rate performance of Zn|CBN@PVDF|PBA cells. (c) The GCD curves of Zn|CBN@PVDF|PBA cells at different rates. (d) Cycling performance and CE of Zn|CBN@PVDF|PBA cells at 0.2 A g^{-1}.

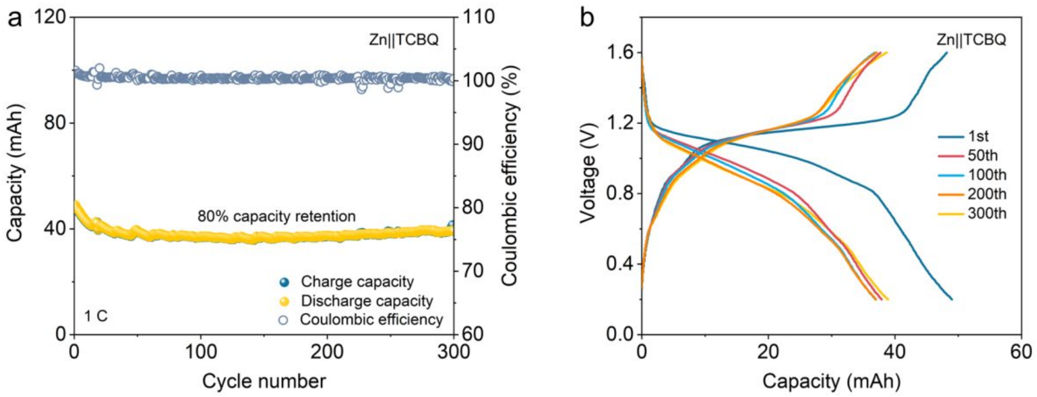

Figure S19. (a) The cycling performance and CE of solid Zn|CBN@PVDF|TCBQ pouch cell at 1 C. (b) The selected GCD curves of solid Zn|CBN@PVDF|TCBQ pouch cell at 1 C.

Table S1 Phase-field simulation parameters.

**Variable name**	**Value of CBN@PVDF**	**Value of PVDF**
CBN concentration in SPE	464.17 mol/m^3	0 mol/m^3
Zn^{2+} bulk concentration in SPE	377.1 mol m^{-3}	377.1 mol m^{-3}
Site density of Zn-metal	109.08 mol m^{-3}	109.08 mol m^{-3}
Conductivity of Zn metal	1.69 × 10^5 S/m	1.69 × 10^5 S/m
Ionic conductivity of SPE	0.00167 S/m	0.0004 S/m
Zn^{2+} diffusion coefficients in the electrode	1.48 × 10^{-13} m^2/s	0.28 × 10^{-13} m^2/s
Solid zinc molar volume	9.572 cm^3/mol	9.572 cm^3/mol
Interfacial tension	1.716 J m^{-2}	1.716 J m^{-2}
Thickness of the solid electrode-electrolyte interface	1× 10^{-6} m	1× 10^{-6} m
Operating current density	1.0 mA cm^{-2}	1.0 mA cm^{-2}
Gas constant	8.3143 J/(mol K)	8.3143 J/(mol K)
Faraday constant	96500 C/mol	96500 C/mol
Temperature	298.15 K (25 °C)	298.15 K (25 °C)

Table S2. Comparison on the ionic conductivity achieved in this work versus previous literature reports.

Solid electrolytes	Testing temperature	Ionic conductivity	Ref.
PVHF/MXene	85 ℃	1.07 mS cm^{-1}	1
PEO/Zn(OTf)$_2$	50 ℃	0.05 mS cm^{-1}	2
PVHF/Zn(OTf)$_2$	25 ℃	0.024 mS cm^{-1}	3
Zn/MOF	30 ℃	0.21 mS cm^{-1}	4
MOF	30 ℃	0.68 mS cm^{-1}	5
PSPZ	25 ℃	0.446 mS cm^{-1}	6
PSIC	25 ℃	0.54 mS cm^{-1}	7
CBN@PVDF	25 ℃	1.67 mS cm^{-1}	This work

Abbreviations:

PVHF/MXene: the poly(vinylidene fluoride-co-hexafluoropropylene) grafted MXenes; PEO: poly ethylene oxide; PVHF: poly (vinylidene fluoride-hexafluoropropylene); MOF: metal organic frameworks; PSPZ: PAN/SiO$_2$-PEO/Zn(OTf)$_2$; PSIC: polymeric single-ion conductors

Table S3. Comparison on the low-temperature Zn reversibility achieved in this work versus previous literature reports tested in Zn||Cu cell configurations.

Solid electrolytes	Cycle time	Polarization voltage	Ref.
PVHF/MXene	0.1 mA/cm^2, 0.1 mAh/cm^2 for 1200 h;<br>0.2 mA/cm^2, 0.2 mAh/cm^2 for 1200 h;<br>0.5 mA/cm^2, 0.5 mAh/cm^2 for 1000 h;	50 mV;<br>101 mV;<br>135 mV	1
ZES	0.1 mA/cm^2, 0.1 mAh/cm^2 for 500 h;<br>1 mA/cm^2, 1 mAh/cm^2 for 100 h;	38 mV;<br>50 mV	8
Zn/MOF	0.1 mA/cm^2, 0.1 mAh/cm^2 for 350 h	120 mV	4
Gel-SA	0.1 mA/cm^2, 0.1 mAh/cm^2 for 1580 h	100 mV	9
PSIC	0.2 mA/cm^2, 0.2 mAh/cm^2 for 2000 h;<br>0.5 mA/cm^2, 0.5 mAh/cm^2 for 1500 h;	70 mV;<br>90 mV	7
CBN@PVDF	0.5 mA/cm^2, 0.5 mAh/cm^2 for 2000 h;<br>1 mA/cm^2, 1 mAh/cm^2 for 1500 h	23 mV;<br>28 mV	This work

Abbreviations:

ZES: zinc bis(trifluoromethylsulfonyl)imide ($Zn(TFSI)_2$)-based eutectic solvent; Gel-SA: gelatin/sodium alginate-acetate

References

1. Z. Chen, X. Li, D. Wang, Q. Yang, L. Ma, Z. Huang, G. Liang, A. Chen, Y. Guo, B. Dong, X. Huang, C. Yang, C. Zhi, Grafted MXene/polymer electrolyte for high performance solid zinc batteries with enhanced shelf life at low/high temperatures. *Energy Environ. Sci.* **2021**, *14*, 3492.
2. R. H. a. G. C. F. H. YANG, CONDUCTIVITY IN PEO-BASED Zn(II) POLYMER ELECTROLYTES. *Solid State Ion.* **1990**, *40*, 663.
3. J. Liu, Z. Khanam, R. Muchakayala, S. Song, Fabrication and characterization of Zn-ion-conducting solid polymer electrolyte films based on PVdF-HFP/Zn(Tf)2 complex system. *J. Mater. Sci.* **2020**, *31*, 6160.
4. Z. Wang, J. Hu, L. Han, Z. Wang, H. Wang, Q. Zhao, J. Liu, F. Pan, A MOF-based single-ion Zn2+ solid electrolyte leading to dendrite-free rechargeable Zn batteries. *Nano Energy* **2019**, *56*, 92.
5. Z. Wang, H. Chen, H. Wang, W. Huang, H. Li, F. Pan, In Situ Growth of a Metal–Organic Framework-Based Solid Electrolyte Interphase for Highly Reversible Zn Anodes. *ACS Energy Lett.* **2022**, *7*, 4168.
6. Y. Li, X. Yang, Y. He, F. Li, K. Ouyang, D. Ma, J. Feng, J. Huang, J. Zhao, M. Yang, Y. Wang, Y. Xie, H. Mi, P. Zhang, A Novel Ultrathin Multiple-Kinetics - Enhanced Polymer Electrolyte Editing Enabled Wide - Temperature Fast - Charging Solid - State Zinc Metal Batteries. *Adv. Funct. Mater.* **2023**, *34*, 2307736.
7. Z. Chen, T. Wang, Y. Hou, Y. Wang, Z. Huang, H. Cui, J. Fan, Z. Pei, C. Zhi, Polymeric Single-Ion Conductors with Enhanced Side-Chain Motion for High-Performance Solid Zinc-Ion Batteries. *Adv. Mater.* **2022**, *34*, e2207682.
8. H. Qiu, X. Du, J. Zhao, Y. Wang, J. Ju, Z. Chen, Z. Hu, D. Yan, X. Zhou, G. Cui, Zinc anode-compatible in-situ solid electrolyte interphase via cation solvation modulation. *Nat. Commun.* **2019**, *10*, 5374.
9. C. Tian, J. Wang, R. Sun, T. Ali, H. Wang, B. B. Xie, Y. Zhong, Y. Hu, Improved Interfacial Ion Migration and Deposition through the Chain-Liquid Synergistic Effect by a Carboxylated Hydrogel Electrolyte for Stable Zinc Metal Anodes. *Angew. Chem. Int. Ed.* **2023**, *62*, e202310970.
